# USP4-mediated CENPF deubiquitylation regulated tumor metastasis in colorectal cancer

**DOI:** 10.1038/s41419-025-07424-3

**Published:** 2025-02-08

**Authors:** Zhongdong Xie, Hanbin Lin, Yuecheng Wu, Yanan Yu, Xintong Liu, Yating Zheng, Xiaojie Wang, Jiashu Wu, Meifang Xu, Yuting Han, Qiongying Zhang, Yu Deng, Lin Lin, Yan Linzhu, Li Qingyun, Xinjian Lin, Ying Huang, Pan Chi

**Affiliations:** 1 Department of Colorectal Surgery, Union Hospital, Fujian Medical University, Fuzhou, China; 2 Department of Colorectal Surgery, The First Affiliated Hospital of Wenzhou Medical University, Wenzhou, China; 3 Central Laboratory, Affiliated Hospital of Putian University, Putian, China; 4 Key Laboratory of Gastrointestinal Cancer (Fujian Medical University), Ministry of Education, Fuzhou, China; 5 Guilin Medical University, Guilin, China; 6 Central Laboratory, The First Affiliated Hospital of Wenzhou Medical University, Wenzhou, China; 7 Department of Science and Technology, The First Affiliated Hospital of Wenzhou Medical University, Wenzhou, China; 8 Department of Pathology, Union Hospital, Fujian Medical University, Fuzhou, China; 9 Department of Pathology, The First Affiliated Hospital of Wenzhou Medical University, Wenzhou, China

**Keywords:** Colorectal cancer, Prognostic markers

## Abstract

Metastasis is a major challenge for colorectal cancer (CRC) treatment. In this study, we identified autophagy activation as a prognostic indicator in CRC and observed that the expression of key autophagy proteins is elevated in metastatic and recurrent cases. Our subsequent goal was to identify potential genes associated with the autophagy panel and assess their prognostic significance, biological roles, and mechanisms in CRC metastasis. Among the candidates, CENPF emerged as the top gene in our screening process. We found that CENPF expression was preferentially elevated in CRC tissues compared to adjacent normal tissues, with significantly higher levels in CRC patients with tumor recurrence. Furthermore, a multicenter cohort study demonstrated that upregulated CENPF expression was strongly associated with poorer disease-free survival in CRC. Functional experiments showed that CENPF knockdown inhibited CRC cell invasion and metastasis both in vitro and in vivo. Intriguingly, we found CENPF undergoes degradation in CRC via the ubiquitination-proteasome pathway. Mechanistically, we observed that USP4 interacted with and stabilized CENPF via deubiquitination. Furthermore, USP4-mediated CENPF upregulation was critical regulators of metastasis of CRC. Examination of clinical samples confirmed that USP4 expression positively correlates with CENPF protein expression, but not mRNA transcript levels. Taken together, this study describes a novel USP4-CENPF signaling axis which is crucial for CRC metastasis, potentially serving as a therapeutic target and a promising prognostic biomarker for CRC.

## Introduction

Colorectal cancer (CRC) is the second most common malignancy worldwide and ranks as the third leading cause of cancer-related deaths [1]. Despite the progress in treatment modalities, CRC recurrence remains a significant concern due to various factors such as tumor heterogeneity, microenvironmental influences, and acquired drug resistance. The recurrence of CRC often signifies a more aggressive disease phenotype, posing a considerable threat to patient survival. A thorough understanding of the molecular processes underlying CRC metastasis has the potential to reveal a plethora of new novel biomarkers and therapeutic targets.

The major type of genomic instability was chromosomal instability (CIN), which was observed in both pre-cancerous lesions and malignant growth [2]. CIN was characterized by frequent chromosomal abnormalities, including whole chromosome or large segmental gains/losses (non-diploidy), structural rearrangements, and focal aberrations (e.g., amplifications and deletions), resulting in tumor heterogeneity and other malignant features. The centromeres and their associated kinetochores were necessary for proper spindle attachment, chromosome alignment, mitotic checkpoint activation, and separation of sister chromatids during mitosis, which were considered major causes of CIN when abnormally expressed. Centromere proteins (CENPs) family participates in centromere formation and organization during mitosis. Centromere protein F (CENPF), one of the CENPs, is the largest member of the centromere protein family (approximately 350 kDa in molecular weight) and plays critical roles in protein complexes, participating in microtubule functions such as attachment and dynamics, centromere assembly, and mitotic checkpoints [3]. Recently, CENPF has been confirmed to potentially induce CIN in primary breast cancer and participate in the progression of various malignant tumors, making it a key factor in tumor progression and a promising indicator for prognosis [4–7]. However, the biological roles and prognostic value of CENPF in CRC remained unknown.

Ubiquitination, a vital posttranslational modification, orchestrates numerous cellular processes, such as cell-cycle advancement, transcriptional modulation, and signal transduction [8–10]. This process involves the attachment of ubiquitin molecules to target proteins, marking them for various fates within the cell. However, alongside ubiquitination, the reversal process known as deubiquitination, managed by deubiquitinating enzymes (DUBs), has emerged as a pivotal regulatory mechanism governing protein turnover [11, 12]. Deubiquitination, in essence, acts as a molecular undo button, removing ubiquitin molecules from target proteins and thereby influencing their stability, activity, and localization within the cell. In recent years, targeting DUBs to regulate ubiquitination modification of substrate proteins has become a hot research direction, and targeting DUBs is emerging as a promising agent for anticancer therapy [13, 14]. Previous studies have shown that several members of the CENP family [15–17] are regulated via the ubiquitin-proteasome pathway. Based on this, we investigated specific deubiquitinating enzymes (DUBs) that regulate CENPF protein expression and further explored the role and potential mechanisms of targeting the DUB-CENPF axis for CRC treatment.

In this research, we employed bioinformatics approaches and conducted siRNA functional screening to identify CENPF as a pivotal gene involved in CRC metastasis. We utilized both in vitro and in vivo CRC cell models to investigate the functional role of CENPF. Additionally, we also evaluated the prognostic relevance of CENPF in CRC by analyzing data from multiple patient cohorts across three different medical centers. Furthermore, our study revealed that CENPF serves as a novel substrate for the Ub-specific protease (USP) family member USP4, a deubiquitinating enzyme closely associated with numerous oncogenic proteins [18–25]. Notably, the interaction between CENPF and USP4 is intricately linked to CRC metastasis.

## Materials and Methods

### Ethics statement

Ethical approval was obtained from the institutional review boards at each participating center (KY2021-R019, 2023KY251) for the retrospective analysis of anonymized data. All procedures involving animals were conducted in accordance with institutional ethical standards for animal experimentation and were approved by the Ethics Committee of Fujian Medical University/Laboratory Animal Center (IACUC FJMU 2022-0488).

### Study patients

The study enrolled a total of 519 patients diagnosed with typical colorectal adenocarcinoma histology. Formalin-fixed, paraffin-embedded (FFPE) specimens were collected from 393 patients who underwent curative surgery at the First Affiliated Hospital, Wenzhou Medical University (Wenzhou, China) from April 2014 to December 2016, constituting Cohort I for the training dataset. Additionally, 126 patients who underwent curative surgery at Union Hospital, Fujian Medical University (Fuzhou, China) from January 2010 to December 2011 formed Cohort II for external validation. Detailed information on the inclusion and exclusion criteria, along with details of the recruitment process, can be found in Supplemental Fig. 10. The baseline characteristics of these patients are summarized in Supplemental Table 1. The retrospective study was approved by the ethics committees of the two centers involved, and the informed consent requirement was waived.

### Follow-up and survival analysis

Patients included in the study underwent follow-up examinations every 3 months during the initial 2 years post-surgery, followed by 6-month intervals for the subsequent 3 years. The last recorded follow-up data were available until September 6, 2021, for patients from Cohort I and until September 1, 2017, for patients from Cohort II. Survival analysis was conducted on patients with complete immunohistochemistry (IHC) data. To determine the optimal cutoff point for epithelial CENPF IHC scores, X-tile software was utilized to establish this cutoff, which correlates CENPF protein expression with DFS. The same threshold values (CENPF IHC score = 170) were then applied to the independent external validation cohorts. Survival analyses were conducted to evaluate the potential relationship between CENPF protein expression and OS and DFS. Additionally, the optimal cutoff values (IHC Score=100) for USP4 were also determined using X-tile software, followed by subsequent survival analysis.

### Immunohistochemistry

Immunohistochemistry (IHC) was performed at the Pathology Laboratory of Union Hospital using rabbit polyclonal antibodies to USP4 (1:1000, ab236987, Abcam) and rabbit polyclonal antibodies to CENPF (1:500, ab5, Abcam), Beclin-1 (1:500, ab62557, Abcam), LC3B (1:400, 3868, CST), ATG7 (1:500, ab52472, Abcam) following the manufacturer’s instructions. IHC scores were calculated by multiplying two components: the intensity score and the extent score. The intensity score is assigned based on a scale (0 for no staining, 1 for weak, 2 for moderate, and 3 for strong), while the extent score represents the percentage of cells showing positive staining. Before evaluating the staining intensity of each protein, a reference standard was set according to the baseline staining levels of each protein. Two experienced pathologists then assess the target protein scores in each patient’s CRC tissue using the established grading system [26], with both pathologists blinded to the sample information. The reliability of the scoring system was evaluated by analyzing the agreement between the scoring results obtained by the two independent investigators using contingency tables (Supplemental Tables 2,3). Cohen’s Kappa Indices were calculated to measure inter-rater agreement, with a result considered to indicate excellent concordance if the Cohen’s Kappa index exceeded 0.8 [27].

### Genomic data mining

Data from GSE14333, GSE41258, and TCGA datasets were obtained from the Gene Expression Ominous (GEO) and TCGA. Then, we extracted corresponding autophagy genes (Supplemental Table 4) expression profile and protein-coding genes from 3 dataset, respectively. In the R 3.2.0 environment, we iteratively explored the correlation between them to identify a candidate set of autophagy-related genes. In the R 3.2.0 environment, we employed the plyr, reshape2, and ggpubr packages to process the raw data from TCGA dataset and display the expression pattern of CENPF mRNA across various cancers. The limma package was employed to explore differentially expressed genes between colorectal cancer (CRC) and adjacent normal tissues in GSE41258 and GSE49355. Additionally, two datasets (GSE8671, GSE22598) were used to investigate the differential expression of CENPF mRNA between colorectal cancer and adjacent normal tissues.

Survival data including disease-free survival (DFS) were annotated from the GSE14333 dataset. The prognostic relevance of CENPF mRNA expression level was investigated in GSE14333 cohort. The X-tile plot curve was utilized to present the relationship between CENPF mRNA expression and DFS, determining the optimal cut-off value to stratify patients. Patients with CENPF mRNA expression above the cut-off value were categorized into the CENPF-high group, while those below were categorized into the CENPF-low group. The Kaplan-Meier method was employed to examine the relationship between different patient groups and DFS.

### Cell models

Cell lines including HCT116, SW480, DLD1 (human colorectal cancer cell lines), and HEK293T (human embryonic kidney epithelial cell line) were obtained from Punuo Sai Life Sciences & Technology Co., Ltd. in Wuhan, China. Authentication of these cell lines was performed via STR profiling. Culture conditions were as follows: HCT116 and DLD1 cells were cultured in RPMI 1640 medium supplemented with 10% fetal bovine serum (Sigma, Missouri, USA) and penicillin/streptomycin, maintained in a 5% CO2 humidified incubator. SW480 and HEK293T cells were cultured in DMEM supplemented with 10% fetal bovine serum and penicillin/streptomycin, also within a 5% CO2 humidified incubator. All cell lines used in this study were confirmed to be free of mycoplasma contamination.

### Immunoprecipitation and ubiquitination assays

In both experiments, cell lysis was performed using a homemade lysis buffer kept on ice. The lysis buffer contained 20 mM Tris-HCl (pH 7.5), 150 mM NaCl, 1 mM EGTA, 1 mM EDTA, 1% Triton X-100, 2.5 mM Sodium pyrophosphate, 1 mM β-Glycerophosphate, and 1 mM Na3VO4, supplemented with a protease inhibitor cocktail. After centrifugation at 12,000 × *g* for 30 min at 4 °C, 5% of the supernatant lysates were retained as input for future use. For the immunoprecipitation of endogenous proteins, protein A/G agarose (obtained from Santa Cruz Biotechnology, Santa Cruz, USA) was washed thrice with lysis buffer and then incubated with the specified antibody along with the remaining cell lysate supernatant at 4 °C overnight. In the case of immunoprecipitation for exogenously overexpressed proteins, anti-Flag (GNI4510-FG), HA (GNI4510-HA), or Myc (GNI4510-MC) affinity gel (obtained from GNI, Tokyo, Japan) was washed three times with lysis buffer and directly incubated with the remaining cell lysate supernatant at 4 °C overnight. Subsequent Western blot analysis was conducted the following day after washing three times with lysis buffer.

### Transwell migration assay

Tumor cell migration assays were conducted following the manufacturer’s guidelines. Initially, cells were harvested and suspended in serum-free medium. Subsequently, they were seeded onto Transwell inserts at a concentration of 200,000 cells per well. These inserts were then positioned in a lower chamber containing 600 μl of culture media supplemented with 20% FBS. The Transwells were then incubated for 24 h at 37 °C. Following incubation, cells on the interior of the Transwell inserts were eliminated using a cotton swab. The cells that had migrated to the lower surface of the membrane were then fixed using 4% paraformaldehyde and stained with 0.1% crystal violet. Photographs were captured from five randomly selected fields, and the cells were counted to determine the average number of cells that had migrated.

### Tumor growth and liver metastasis assay in nude mice

Five-week-old female athymic nude mice were obtained from the GemPharmatech Co., Ltd (Nanjing, China). After the purchased experimental mice have adapted to the SPF-level barrier for one week, their body weight will be measured. Based on the body weight, the mice will be randomly assigned into the experimental group and the control group. The mice (*n* = 5 per group) were subcutaneously injected with either 3 × 10^6^ DLD-1 cells or SW480 cells or HCT116 cells transduced with lenti-shCtrl or lenti-shCENPF1 or lenti-shCENPF2. Tumor volume (mm^3^) was calculated using the formula: Volume = 0.5 × length × (width)^2^. The investigator was blinded to group assignments throughout the experiment and when evaluating the outcomes.

To assess the metastatic potential of colorectal cancer (CRC) cells in the liver, athymic nude mice (*n* = 6 per group) were utilized, following established protocols. Briefly, a small incision was made in the left abdomen, and the spleen was isolated and exposed. Viable cancer cells (3 × 10^6^ cells/50 μl PBS) were injected into the spleen using a sterile tuberculin syringe and a 27-gauge needle. Then, the abdominal cavity was closed using nylon sutures. Mice were euthanized after eight weeks (for DLD-1 and SW480 cells), and liver metastases were subsequently evaluated.

### CCK-8 assay, colony formation assay

The viability of cells was assessed using the Cell Counting Kit-8 (CCK-8) from Dojindo Molecular Technologies, Inc. Cells were seeded into triplicate wells of 96-well plates at a density of 2000 cells per well for the CCK-8 assay. For the colony formation assay, cells were cultured in triplicate wells of 6-well plates at a density of 500 cells per well. Following a two-week incubation period under standard growth conditions, the colonies were fixed with ice-cold 4% paraformaldehyde, stained with crystal violet solution, and examined using an inverted microscope.

### Western blot analysis

Western blot analysis for conventional protein was performed as previously described [28]. For the analysis of high molecular weight proteins in this study, we utilized an extended-range prestained protein marker (Proteintech, Wuhan, China) and a specialized gel kit (Epizyme, Shanghai, China). Electrophoresis was conducted at 130 V for 3 h, with the running buffer replaced 1–2 times during the process. For protein transfer, the transfer buffer was supplemented with 0.1% SDS and 10% methanol, and the transfer was carried out on ice at 300 mA for 3 h, with the buffer replaced 1–2 times. All other steps were performed according to the standard Western blot protocol. Antibodies used in Western blot were USP4 (1:1000, ab236987, Abcam), CENPF (1:1000, ab5, Abcam), HA (1:3000, ab236632, Abcam), Flag (1:2000, 14793, CST), Myc (1:3000, AE070, Abclonal), GAPDH (1:5000, 60004-1-Ig, Proteintech), β-actin (1:3000, ab8226, Abcam).

### Immunofluorescence (IF)

Immunofluorescence staining was carried out as described previously [28]. In brief, Cells were cultured on 35 mm glass-bottom dishes (Thermo Scientific™, Massachusetts, USA), fixed in 4% paraformaldehyde for 15 min, and blocked with 5% goat serum in PBS with 0.3% Triton X-100 (Sigma, Missouri, USA). They were incubated overnight with CENPF (1:400, ab5, Abcam) and USP4 (1:200, 66822-1-Ig, Proteintech) primary antibodies, followed by Alexa Fluor 488 anti-Rabbit and Alexa Fluor 594 anti-Mouse secondary antibodies. Nuclei were stained with DAPI (Roche, Basel, Switzerland) and observed using Leica SP5 fluorescence microscopy.

### Statistical analysis

Statistical analyses were performed using R (version 3.5.0) and SPSS (version 16.0.2). Significance was set at *p* < 0.05 for all two-tailed tests. Experiments included 3 to 10 samples per group, with results reported as mean ± SE from at least 3 independent experiments. The Shapiro-Wilk test assessed data normality. For normally distributed data, independent sample *t*-tests compared two groups, and one-way ANOVA compared three or more groups. Pearson’s correlation tested relationships between variables. For non-normally distributed data, the Mann–Whitney test compared two groups, Kruskal-Wallis test compared three or more groups, and Spearman’s rank correlation tested relationships. Two-way ANOVA analyzed repeated measurements. Clinical characteristic comparisons used Mann–Whitney U, Fisher’s exact, or chi-square tests. X-tile software optimized cutoff values for marker expression subgroups. Kaplan-Meier analysis and log-rank tests estimated overall and disease-free survival, while multivariate Cox regression assessed marker contributions to survival outcomes.

## Results

### Identification of autophagy-related CENPF as a key participant in CRC metastasis

**Fig. 1:**
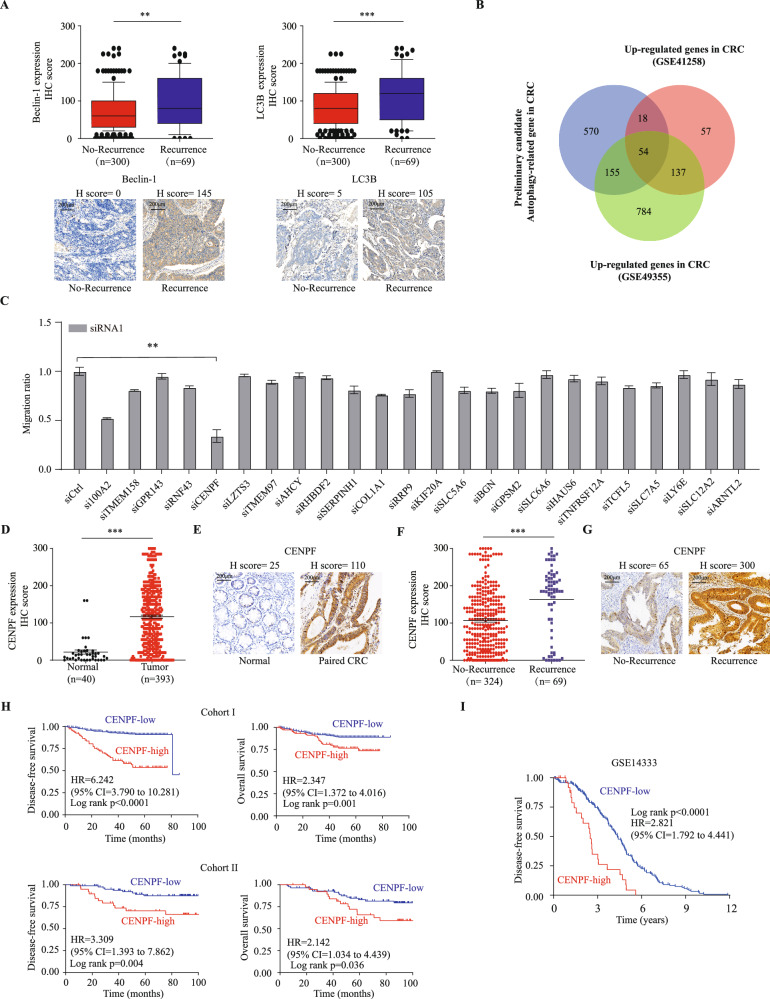
Multifaceted data analysis identified CENPF as a top candidate gene involved in CRC progression. **A** Significant upregulation of Beclin-1, LC3B protein expression was observed in CRC with CRC recurrence compared to CRC without CRC recurrence. Evaluation based on the nonparametric Mann–Whitney test. Significance indicated by **P < 0.01, ***P < 0.001. Scale bar, 200 μm. **B** Venn diagram demonstrates the intersection of initial candidate autophagy-related genes with differentially expressed genes from GSE41258 and GSE49355 datasets. **C** HCT116 cells were subjected to treatment with siRNA1 targeting candidate genes, and transwell assays were conducted to evaluate the migration of each cell group. Migration rates were calculated as the ratio of each cell group to the control, and bar graphs depict the migration rates for each group. Results were analyzed using the Student’s *t* test. **p < 0.01. **D** A scatter plot displays the IHC expression scores of CENPF protein in normal tissues compared to colorectal cancer tissues from patients in cohort I. The Mann–Whitney non-parametric test was used to compare the expression differences, with the *p* value indicated in the graph. **E** Representative schematic diagram of immunohistochemical staining for CENPF protein in colorectal epithelial tissues. The scoring of representative images is shown in the figure. “Normal” refers to normal tissue; “Paired CRC” refers to paired colorectal cancer tissue. Scale bar, 200 μm. **F** Immunohistochemistry analysis of CENPF protein levels in CRC with recurrence versus without recurrence in Cohort I. ***p < 0.001. **G** Representative schematic diagram of immunohistochemical staining for CENPF protein in CRC with recurrence compared to without recurrence in Cohort I. Scale bar, 200 μm. **H** Kaplan–Meier survival curves representing overall survival (OS) and disease-free survival (DFS) of CRC patients, determined by their levels of CENPF protein expression in Cohort I (*n* = 393) and Cohort II (*n* = 126). The log-rank test was used to compare the differences between the survival curves of the two groups, with *p* values shown in the graphs. **I** Kaplan–Meier analysis was performed to explore the relationship between CENPF mRNA expression and disease-free survival in the GSE14333 cohort (*n* = 226). The log-rank test was used to compare the differences between the survival curves of the two groups, with the *p* value shown in the graph.

Autophagy is considered a self-degradative and conservative process [29], playing a crucial role in controlling the quality of cellular components and maintaining cellular homeostasis. Recently, it has been found that autophagy not only supports tumor growth in harsh environments but also plays a critical role in tumor metastasis [30–37]. To explore the involvement of autophagy in CRC metastasis, we analyzed the expression patterns and clinical relevance of key autophagy regulators in colorectal cancer tissues. Our findings revealed that the expression levels of Beclin-1, LC3B, and ATG7 proteins in epithelial cells progressively increased from adjacent normal tissues to primary tumors, and were further elevated in metastatic cancers (all *P* < 0.001; Supplemental Fig. 1A). Moreover, significant upregulation of these autophagy proteins was observed in CRC cases with recurrence compared to those without recurrence (all *P* < 0.01; Fig. 1A, Supplemental Fig. 1B). Survival analysis of CRC patients from cohort I with follow-up data indicated that high expression of Beclin-1, LC3B, and ATG7 was associated with shorter disease-free survival (*P* < 0.0001; Supplemental Fig. 1C). These results suggest that autophagy activation may contribute to CRC metastasis. Building on these observations, we further explored gene expression data from biobank databases to identify genes potentially involved in CRC metastasis and correlated with the autophagy gene set. We initially obtained raw data from GSE14333, GSE41258, and TCGA gene sets, extracted the expression profile of autophagy genes and the remaining protein-coding genes, and performed correlation analysis on both sets. The potential autophagy-related gene sets (correlation > 0.3 and *p* < 0.001) were obtained for GSE14333, GSE41258, and TCGA gene sets, and their intersection was taken as the initial candidate set of 797 autophagy-related genes (Supplemental Fig. 1D). We then compared those candidates with a group of genes that were highly expressed (logFC > 1 and fdr < 0.0001) in GSE41258 and GSE49355 datasets, resulting in 54 potential important CRC-related genes (Fig. 1B). We further narrowed down the candidate genes based on single-cell databases, literature searches to exclude stromal or immune cell localization, and previous studies on genes in CRC, focusing on 24 candidate genes that may play a role in CRC. We obtained two small interfering RNAs (siRNAs) targeting each of the 24 genes and a control siRNA (siCtrl), then transfected into HCT116 CRC cells separately, and evaluated the impact on the migration ability of HCT116 cells using migration assays. It was found that knocking down CENPF most significantly inhibited the migration of HCT116 cells (Fig. 1C and Supplementary Fig. 1E).

Also, we analyzed published CRC mRNA microarray datasets from GSE8671, GSE22598 and TCGA. In TCGA, we observed that the expression of CENPF mRNA is significantly elevated in colorectal cancer and rectal cancer, similar to many other broad-spectrum tumors, compared to adjacent normal tissues (Supplemental Fig. 2A). This finding is further validated by two additional datasets, GSE8671 and GSE22598, confirming the upregulation of CENPF expression in CRC (Supplemental Fig. 2B, C). Additionally, we have collected an independent cohort of tissues microarrays (TMAs) composed of colorectal cancer and adjacent normal tissues (Cohort I). We examine the expression of CENPF protein using immunohistochemistry staining. We observed CENPF protein was mainly distributed in both cytoplasm and nucleus of colorectal epithelial cell as representative immunostaining presented (Fig. 1E), in which the expression pattern of CENPF protein mirrors that of mRNA expression (Fig. 1D). Likewise, CENPF protein expression was significantly higher in CRC patients with tumor recurrence (Fig. 1F and Supplementary Fig. 2D) as demonstrated by representative immunostaining (Fig. 1G). Thus, these findings suggest that CENPF may play a critical role in CRC metastasis.

To assess the potential prognostic significance of dysregulated CENPF expression in CRC, we then conducted time-to-event analyses using 3 cohorts with follow-up records. In Cohort I, high CENPF protein expression was associated with more advanced TNM stage (*p* = 0.002) (Supplementary Table 1). Importantly, high CENPF protein expression was correlated with poorer DFS and OS when compared to the low-expression group (HR 6.242, 95% CI 3.790 to 10.281, Log-rank p < 0.0001; HR 2.347, 95% CI 1.372 to 4.016, Log-rank *p* = 0.001, respectively) (Fig. 1H). Furthermore, multivariate Cox regression analysis demonstrated that high CENPF protein expression was an independent predictor for both DFS and OS in CRC patients (HR 5.282, 95% CI 3.162 to 8.822, *p* < 0.001; HR 2.074, 95% CI 1.192 to 3.607, *p* = 0.01, respectively) (Table 1). To ensure the reliability and applicability of these results, we conducted analogous examinations within an independent external validation cohort. Consistently, high CENPF protein expression was linked to poorer DFS and OS compared to the low-expression group in Cohort II (HR 3.309, 95% CI 1.393 to 7.862, Log-rank *p* = 0.004; HR 2.142, 95% CI 1.034 to 4.439, Log-rank *p* = 0.036, respectively) (Fig. 1H). Further validation through multivariate Cox regression analyses consistently demonstrated that elevated CENPF protein expression remained a strong and independent prognostic factor for both DFS and OS among CRC patients within the external validation cohort II (Table 2). In GSE14333, it was found that higher CENPF expression in tumors was significantly associated with decreased DFS (HR 2.821, 95% CI 1.792 to 4.441, Log-rank *p* < 0.0001) (Fig. 1I). Collectively, these findings indicate a tumor-promoting role of CENPF in CRC.

**Table 1:** Cox regression analysis of CENPF protein expression and clinicopathological covariates with survivals in the Cohort I.

	Disease-free Survival				Overall Survival			
	Univariate analysis		Multivariate analysis		Univariate analysis		Multivariate analysis	
	HR (95%CI)	*p* Value	HR (95%CI)	*p* Value	HR (95%CI)	*p* Value	HR (95%CI)	*p* Value
CENPF-high *vs*. CENPF-low	6.242 (3.790–10.281)	<0.001	5.282 (3.162–8.822)	<0.001	2.347 (1.372–4.016)	0.002	2.074 (1.192–3.607)	0.010
Age (≥60 vs. < 60)	1.617 (0.959–2.724)	0.071			1.125 (0.647–1.955)	0.676		
Sex (male *vs*. female)	0.933 (0.570–1.528)	0.783			0.797 (0.463–1.371)	0.412		
Location (rectum vs colon)	1.292 (0.806–2.071)	0.288			0.714 (0.411–1.241)	0.233		
TNM stage (III + IV vs I + II)	4.731 (2.829–7.911)	<0.001	3.192 (1.818–5.605)	<0.001	3.483 (1.992–6.091)	<0.001	2.050 (1.097–3.832)	0.025
Differentiation grade (poorly vs others)	2.102 (1.213–3.640)	0.008	1.260 (0.678–2.344)	0.465	2.325 (1.282–4.218)	0.005	1.094 (0.555–2.156)	0.795
Adjuvant chemotherapy (yes vs no)	1.912 (1.104–3.310)	0.021	1.130 (0.635–2.012)	0.678	0.698 (0.409–1.192)	0.188		
Lymphovascular invasion (yes vs no)	1.887 (1.031–3.456)	0.040	0.793 (0.406–1.547)	0.496	2.779 (1.512–5.109)	0.001	1.446 (0.732–2.860)	0.289
Perineural invasion (yes vs no)	2.461 (1.319–4.592)	0.005	1.770 (0.866–3.618)	0.118	2.514 (1.264–4.998)	0.009	1.942 (0.864–4.366)	0.108
Tumor size (cm) (≥4 vs <4)	1.319 (0.819–2.124)	0.254			2.172 (1.262–3.738)	0.005	2.046 (1.156–3.621)	0.014
Serum CEA (ng/ml) (≥5 vs <5)	1.651 (1.029–2.649)	0.038	1.158 (0.683–1.965)	0.586	2.206 (1.290–3.774)	0.004	1.317 (0.725–2.392)	0.366
Serum CA199 (U/ml) (≥37 vs <37)	2.024 (1.118–3.663)	0.020	1.553 (0.808–2.988)	0.187	3.382 (1.884–6.069)	<0.001	2.141 (1.118–4.099)	0.022

*HR* hazard ratio, *CI* confidence interval.

**Table 2:** Cox regression analysis of CENPF protein expression and clinicopathological covariates with survivals in the Cohort II.

	Disease-free Survival				Overall Survival			
	Univariate analysis		Multivariate analysis		Univariate analysis		Multivariate analysis	
	HR (95%CI)	*p* Value	HR (95%CI)	*p* Value	HR (95%CI)	*p* Value	HR (95%CI)	*p* Value
CENPF-high vs. CENPF-low	3.309 (1.393–7.862)	0.007	3.991 (1.635–9.738)	0.002	2.142 (1.034–4.439)	0.040	2.651 (1.225–5.738)	0.013
Age (≥60 vs. < 60)	1.699 (0.659–4.383)	0.273			1.886 (0.835–4.262)	0.127		
Sex (male *vs*. female)	0.817 (0.339–1.973)	0.654			1.980 (0.806–4.865)	0.136		
Location (rectum vs colon)	1.043 (0.574–1.894)	0.890			1.496 (0.913–2.452)	0.110		
TNM stage (III + IV vs I + II)	2.021 (1.135–3.596)	0.017	2.544 (1.360–4.758)	0.003	3.566 (2.141–5.940)	<0.001	3.751 (2.131–6.600)	<0.001
Differentiation grade (poorly vs others)	1.502 (0.714–3.163)	0.284			1.619 (0.865–3.032)	0.132		
Adjuvant chemotherapy (yes vs no)	2.101 (0.770–5.735)	0.147			1.347 (0.613–2.959)	0.458		
Tumor size (cm) (≥4 vs <4)	0.436 (0.183–1.039)	0.061			0.483 (0.231–1.013)	0.054	0.907 (0.395–2.081)	0.817
Serum CEA (ng/ml) (≥5 vs <5)	2.492 (1.032–6.018)	0.042	1.696 (0.650–4.430)	0.280	1.868 (0.897–3.889)	0.095	1.740 (0.831–3.642)	0.142
Serum CA199 (U/ml) (≥37 vs <37)	3.268 (1.352–7.895)	0.009	3.239 (1.197–8.763)	0.021	1.706 (0.727–4.000)	0.219		

*HR* hazard ratio, *CI* confidence interval.

### Knockdown of CENPF inhibits migration, invasion of colorectal cancer cells in vitro and in vivo

**Fig. 2:**
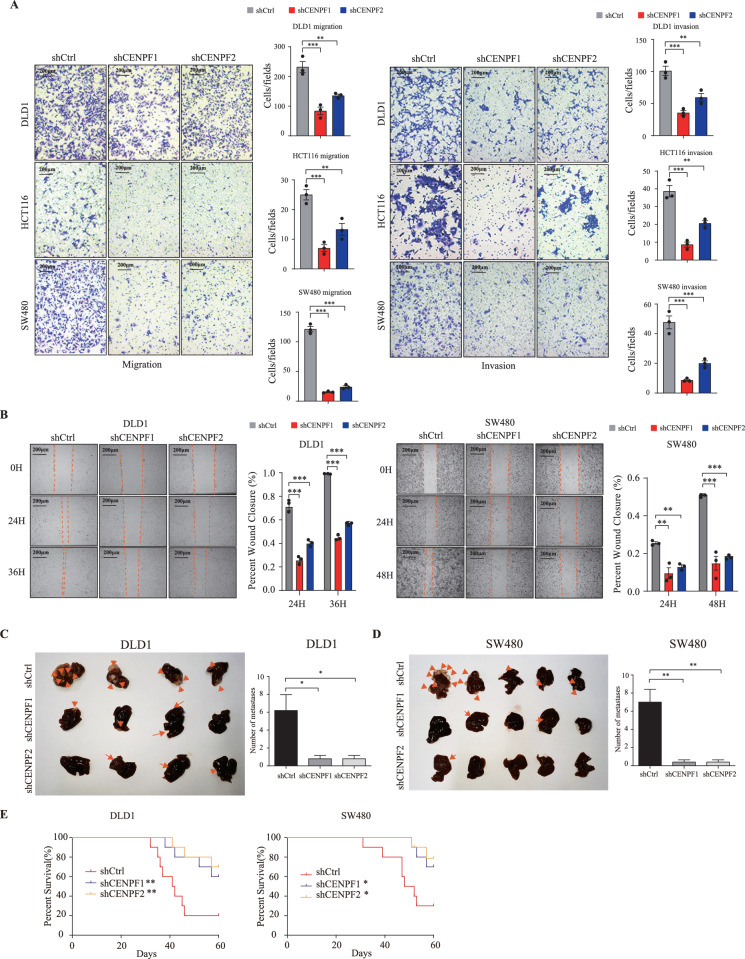
Knockdown of CENPF inhibits colorectal cancer cell migration, invasion in vitro and in vivo. **A** Transwell assays were performed to evaluate migration and invasion ability of following CENPF knockdown in DLD1, HCT116, SW480 cells. Scale bars, 200 μm. The experiment was repeated three times. One-way ANOVA was used to compare the differences in the number of cells invading through the migration chamber in different groups. **p < 0.01; ***p < 0.001. **B** Representative images of the wound-healing migration assays in CENPF-knockdown DLD1 and SW480 cells compare with shCtrl cells. Scale bars, 200 μm. One-way ANOVA was used to compare the differences in the wound healing area at different time points. **p < 0.01, ***p < 0.001. **C**, **D** Representative photographs and quantification of metastatic tumor nodes in mouse livers after spleen injection (intrahepatic metastases were marked with yellow arrows). Differences in liver lesions among groups were analyzed using one-way ANOVA test. *p < 0.05, **p < 0.01. **E** Kaplan–Meier curves showing overall survival among different groups of mice (10 mouse models in each group). Log-rank test was used to calculate *p*-values compared with the shCtrl group. *p < 0.05; **p < 0.01.

The prevalence of CENPF upregulation raises an intriguing possibility that CENPF overexpression may be a cancer-promoting event in CRC. To examine this possibility, CENPF expression was stably knock down using lentiviral vectors carrying two shRNAs targeting CENPF (shCENPF1 and shCENPF2) in DLD-1, HCT116, and SW480 cells. The knockdown efficiency was confirmed by Western blot assays (Supplementary Fig. 3A). Colorectal cancer cell migration and invasion ability was assessed in vitro. The migration and invasion assays showed that knocking down CENPF significantly weakened the ability of colorectal cancer cell migration and invasion (Fig. 2A). Similarly, scratch-wound-healing assays revealed that a lower migration ability in shCENPF cells than in shCtrl cells (Fig. 2B). To further exclude off-target effects of CENPF shRNA in CRC, CENPF-HA overexpression plasmids was transiently transfected into stably CENPF-knockdown DLD1, HCT116, and SW480 cells, examined by Western blot assays (Supplementary Fig. 4A). The migration and invasion assays revealed that CENPF overexpression significantly reversed the reduced migration and invasion compared to the vector group (Supplementary Fig. 4B). Moreover, scratch wound healing assays demonstrated a noticeable restoration of migration capacity in CENPF-overexpressing DLD1 and SW480 cells compared to vectors (Supplementary Fig. 4C). However, CCK-8 assays and Colony assays were used to investigate the role of CENPF in cell proliferation. The results revealed that knockdown of CENPF had no significant effect on CRC cell growth in vitro (Supplementary Fig. 5A, B). Overall, these findings showed that CENPF can enhance the migration, and invasion of colorectal cancer cells in vitro.

We further examine whether the same result can be observed in vivo. CRC cells with or without CENPF knockdown were injected into spleen of the nude BALB/C mice (Supplementary Fig. 3B). Eight weeks post-injection, the ability of SW480 and DLD1 cells to develop liver micrometastases were significantly impaired when cells lacked CENPF expression (Fig. 2C, D). Similarly, histological examination showed that the shCENPF group developed less liver micrometastases (Supplementary Fig. 3C, D). Consistently, CENPF knockdown prolonged mouse survival (Fig. 2E). However, no significant difference in tumor size was observed between CENPF knock-down group and shCtrl group (Supplementary Fig. 5C). Together, these results indicated that CENPF functioned as a tumor promoter in CRC metastasis.

### USP4 interacts with CENPF and is identified as a candidate DUB for CENPF

**Fig. 3:**
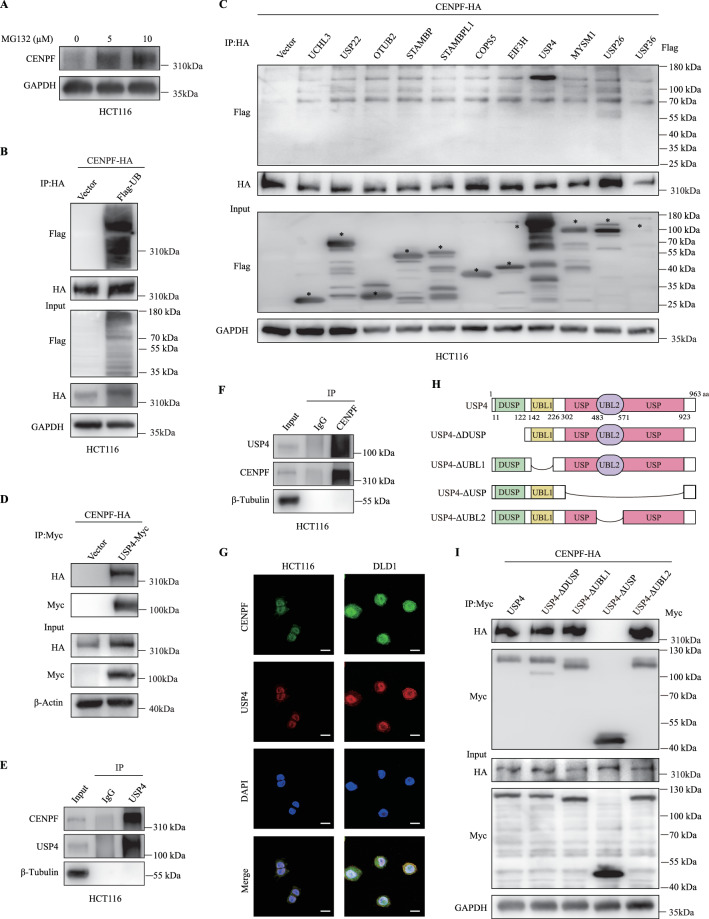
USP4 interacts with CENPF. **A** Western blot analysis comparing CENPF and GAPDH levels in HCT116 cells under MG132 treatments of 0 µM, 5 µM, and 10 µM. **B** HCT116 cells were transfected with expression plasmids CENPF-HA or Vector along with Flag-UB, followed by treatment with 20 µM MG132 for 6 h before harvesting. CENPF-HA was then immunoprecipitated with anti-HA antibody, and ubiquitinated CENPF detected using anti-Flag antibody. **C** Immunoprecipitation (IP, with anti-HA) and immunoblot analysis (with anti-FLAG and anti-HA) of HCT116 cells transfected with plasmids encoding FLAG-tagged DUBs and CENPF-HA for 48 h. **D** Co-immunoprecipitation (Co-IP) was used to analyze the interaction between exogenous CENPF-HA and USP4-Myc. After transfecting HCT116 cells with specific plasmids, USP4-Myc was immunoprecipitated from the lysates and subsequently immunoblotted using the provided antibodies. Cells were pre-treated with 20 µM MG132 for a duration of 6 h. **E**, **F** Co-IP analysis of the endogenous interaction between CENPF and USP4 in HCT116 cells. Cell lysates were immunoprecipitated with an anti-USP4 antibody (**E**) or an anti-CENPF antibody (**F**), and interactions were detected by immunoblotting. **G** Visualization of CENPF and USP4 through confocal microscopy in HCT116 and DLD1 cells, with a scale bar at 20 µm. **H** Schematic representation of USP4 and mutants. **I** HCT116 cells, post co-transfection with CENPF-HA and USP4-Myc or its mutants, were subjected to anti-Myc immunoprecipitation and consequent immunoblotting after a 20 µM MG132 treatment for 6 h.

Previous studies have revealed that several members of the CENP family, such as CENPA [15], CENPH [16], and CENPN [17], are degraded through the ubiquitin-proteasome pathway. Given this, we further investigated whether the CENPF protein follows a similar degradation pathway. Initially, we utilized protein modification site prediction tools and identified several potential ubiquitination sites on the CENPF protein structure (Supplemental Fig. 6A). Western blotting showed CENPF protein increased with MG132 treatment, proteasome inhibitor, in a dose-dependent manner (Fig. 3A). And we performed co-immunoprecipitation (Co-IP) assays by transfecting HCT116 cells with CENPF-HA and Flag-Ubiquitin, and anti-HA immunoprecipitates were probed for the level of ubiquitination using Flag-antibody (Fig. 3B). It was discovered that CENPF undergoes ubiquitination, suggesting its degradation in CRC via the ubiquitination-proteasome pathway.

The proteasome identifies proteins tagged with ubiquitin and breaks them down into smaller peptides and amino acids. Conversely, deubiquitinating enzymes (DUBs) work to remove or cleave ubiquitin molecules from protein substrates, playing a crucial role in numerous cellular processes as a key regulatory mechanism [38]. To identify potential DUBs for CENPF, we screened DUBs expression library consisting of 53 human DUBs in HEK293T cells. HA-tagged CENPF was co-transfected with 53 DUBs-Flag plasmids separately into HEK293T cells, and expression of CENPF was detected using WB 72 h after transfection. After the initial round of screening (Supplementary Fig. 6B), eighteen candidate DUBs that stabilized CENPF level significantly, were tested in the second round of screening in HCT116 CRC cells (Fig. 3C). Among the candidates, USP4 could interact with CENPF. Immunoprecipitation with anti-Myc antibody demonstrated that Myc-USP4 interacted with overexpressed CENPF-HA (Fig. 3D). To determine whether the interaction between USP4 and CENPF also occurs endogenously in CRC cells, immunoprecipitation was performed using anti-USP4 or anti-CENPF antibodies on lysates from HCT116 cells. IP with either antibody resulted in the co-detection of both USP4 and CENPF, indicating that these endogenous proteins interact in CRC cells (Fig. 3E, F). Furthermore, immunofluorescence staining revealed that CENPF (green) and USP4 (red) were colocalized with the nucleus, as indicated by the DAPI stain, in multiple CRC cell lines (Fig. 3G). We then identified which USP4 regions are critically required for its interaction with CENPF. We generated four USP4 truncations (Fig. 3H), and through Co-IP assays, we found that the USP domain mediated association with CENPF (Fig. 3I). Therefore, USP4 could interact with CENPF and markedly stabilized CENPF protein expression levels, which was the most promising candidate based on the overlap of these two screenings.

### USP4 deubiquitinates and stabilizes CENPF

**Fig. 4:**
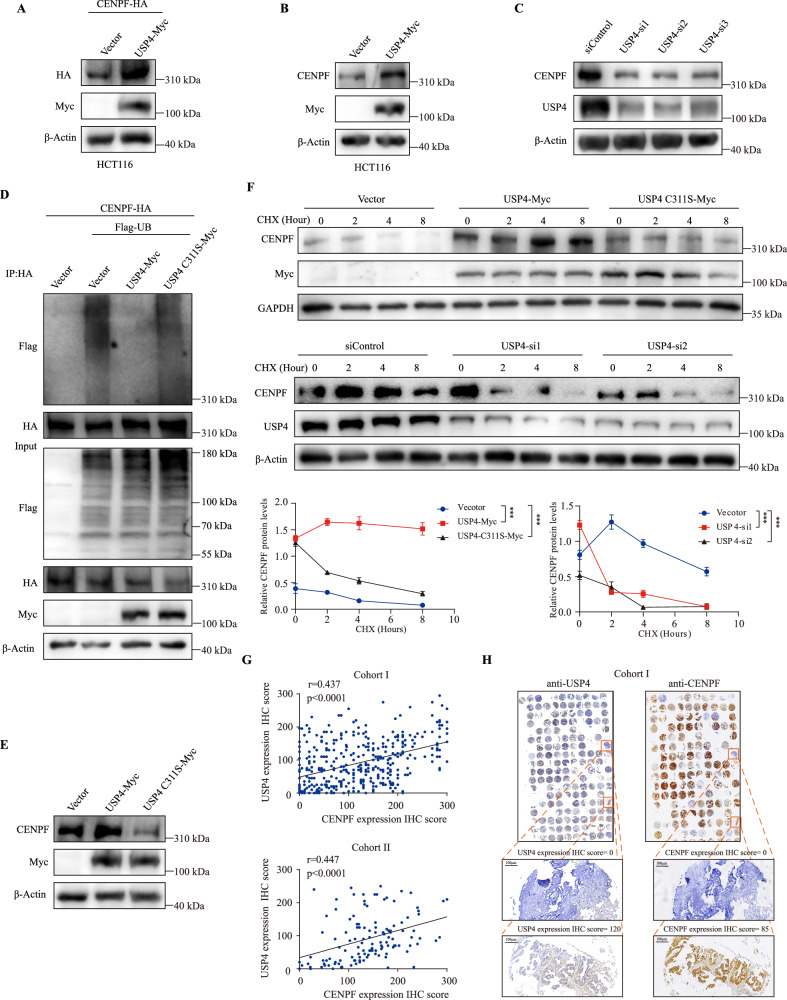
USP4 deubiquitinates and stabilizes CENPF. **A** CENPF-HA expression plasmids were separately transfected into HCT116 cells along with Vector or USP4-Myc plasmids. After 48 h, Western blot analysis was performed to detect the exogenous expression level of CENPF-HA. **B** An immunoblot analysis studying CENPF expression in HCT116 cells post-transfection with USP4-Myc expression plasmid. **C** Immunoblot examination of CENPF levels in HCT116 cells post-transfection with three distinct siRNAs targeting USP4. **D** After co-transfecting HCT116 cells with various constructs and treating with 20 µM MG132 for 6 h, ubiquitinated CENPF was identified using an anti-Flag antibody post-immunoprecipitation. **E** An immunoblot analysis studying CENPF expression in HCT116 cells post-transfection with Vector, USP4-Myc, or the enzymatically inactive mutant USP4 C311S-Myc plasmid. **F** Upper: CENPF-HA expression plasmids were separately co-transfected with Vector, USP4-Myc, or USP4 C311S-Myc expression plasmids into HCT116 cells. After 48 h, cells were treated with 40 µg/mL cycloheximide (CHX) for set period. CENPF-HA’s expression was then scrutinized through a Western blot employing anti-HA antibody. Subsequent normalization of CENPF expression took place against GAPDH levels. Significance determined using Student’s *t* test. **p < 0.01; n.s. signifies non-significant. Lower: HCT116 cells were separately transfected with siRNAs targeting USP4. After 72 h, cells were treated with 40 µg/mL cycloheximide (CHX) for set period. CENPF’s expression was then scrutinized through a Western blot employing anti-CENPF antibody. Subsequent normalization of CENPF-HA or CENPF expression took place against GAPDH or β-Actin levels. Significance determined using Student’s *t* test. ***p < 0.001. **G** Upper: Spearman’s rank correlation analysis was performed based on immunostaining scores of USP4 and CENPF from the cohort I’s CRC tissue microarray (*r* = 0.437, *p* < 0.0001, *n* = 393). Lower: Spearman’s rank correlation analysis was performed based on immunostaining scores of USP4 and CENPF from the cohort II’s CRC tissue microarray (*r* = 0.447, *p* < 0.0001, *n* = 126). **H** Exemplary immunohistochemical stains of CENPF and USP4 in CRC tissues are displayed. Scale bar represents 500 μm.

To further elucidate the role of USP4 in its interaction with CENPF, further investigations demonstrated that USP4 could stabilize endogenously and exogenously expressed CENPF (Fig. 4A, B). USP4 knockdown significantly reduced CENPF protein expression (Fig. 4C). However, neither the overexpression (Supplementary Fig. 7A) nor the knockdown (Supplementary Fig. 7B) of USP4 affected CENPF mRNA levels, indicating that USP4 stabilized CENPF protein levels post-transcriptionally.

USP4 is categorized as a cysteine protease, with bioinformatic analyses pinpointing C311 as the crucial catalytic cysteine. Consequently, USP4 mutant variant was created, wherein a substitution of cysteine to serine at position 311 (C311S) was introduced, rendering the enzyme catalytically deficient. To whether USP4 can indeed deubiquitinate CENPF, HCT116 cells were transfected with CENPF-HA, Flag-UB, USP4-Myc or USP4 C311S-Myc, and then anti-HA immunoprecipitates were probed for the level of ubiquitination using Flag-antibody. Co-expression of USP4 and CENPF led to a notable effect on diminishing ubiquitination of CENPF (Fig. 4D). However, enzyme-inactive mutant USP4 C311S was not able to reduce CENPF ubiquitination (Fig. 4D). Moreover, overexpression of WT USP4, but not the enzyme-inactive mutant USP4 C311S, could stabilize CENPF protein (Fig. 4E). These results suggested that deubiquitylation and stabilization of CENPF was dependent on the catalytic activity of USP4. We next tested whether USP4 indeed extended CENPF’s protein half-life by cycloheximide chase assay. Similarly, overexpression of WT USP4, but not USP4 C311S, prolonged the half-life of CENPF (Fig. 4F). Conversely, when USP4 was depleted from HCT116 cells, the half-life of CENPF was markedly reduced (Fig. 4F). These data demonstrated that USP4 interacts with CENPF and facilitates ubiquitination of CENPF, stabilizing it and prolonging its lifespan when overexpressed. Conversely, depletion of USP4 reduces CENPF’s lifespan.

To confirm the relevance of the USP4-CENPF interaction, we first analyzed protein expression of USP4 and CENPF in our clinical samples from two different Cohort. We found that USP4 and CENPF expression were positively correlated (Fig. 4G). Representative immunostaining results were presented in Fig. 4H. However, public dataset analysis of USP4 and CENPF mRNA levels did not mirror these results (Supplementary Fig. 7C). Notably, a high expression level of USP4 correlated with poor prognosis of CRC patients from two different cohorts (Supplementary Fig. 8A). Taken together, these findings suggested that USP4 was a strong DUB for CENPF, which at least partially, contributed to the upregulated CENPF protein expression.

### CENPF is positively regulated by USP4 and affects the metastatic ability of CRC cells

**Fig. 5:**
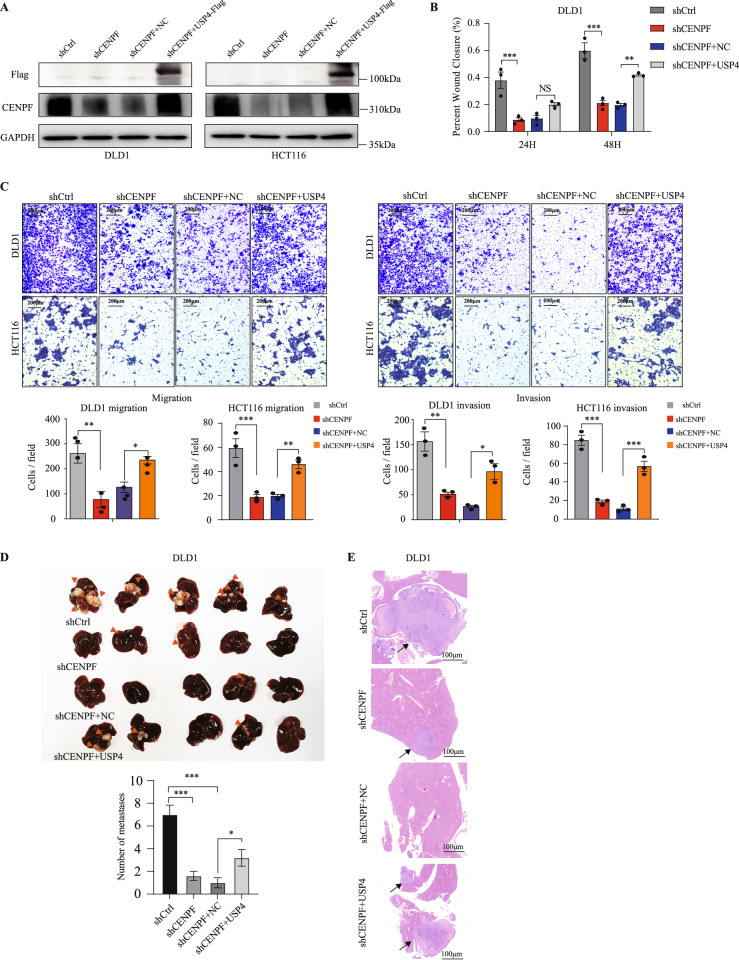
USP4 upregulation alleviates CENPF-Knock down induced CRC migratory capacity and metastasis ability suppression in vitro and in vivo. **A** Western blotting results showing the expression of USP4-Flag, CENPF, and GAPDH proteins from samples of the mentioned cells. **B** Quantification of the wound healing assays using indicated cells. Differences in the area of wound closure among different groups were compared using one-way ANOVA. **p < 0.01, ***p < 0.001. NS, non-significant. **C** Representative images and quantification of the transwell assays using transfected DLD1/HCT116 cells. Differences in the number of cells traversing the migration chamber among different groups were compared using one-way ANOVA. **p < 0.01, ***p < 0.001. **D** Representative photographs and quantification of metastatic tumor nodes in mouse livers after spleen injection (intrahepatic metastases were marked with yellow arrows) using transfected DLD1 cells among different groups. Differences in liver lesions of different groups were analyzed using one-way ANOVA test. *p < 0.05, ***p < 0.001. **E** Representative HE staining images of DLD1 tumors in the 8 weeks after spleen injection among different groups. Scale bars, 100 μm.

After identifying the interaction between USP4 and CENPF, we further examined the effectiveness of USP4 on the CENPF mediated enhanced CRC migration, invasion, and metastasis capacity. In HCT116 and DLD1 cell lines, we knocked down CENPF using shCENPF and stably overexpressed USP4 for subsequent research. Western blot analysis was utilized to detect the levels of USP4 and CENPF in cell lines from various groups, as illustrated in Fig. 5A. The transwell assays and wound healing assays were employed to assess the invasion and migration capabilities. The results showed that overexpressing USP4 significantly enhanced CRC^’^s migratory ability under CENPF-knocking down conditions (Fig. 5B, C and Supplementary Fig. 9). Additionally, we investigated whether similar results could be observed in vivo. These findings were reinforced by conducting an animal study, where DLD1 cells were injected into the spleen of nude mice using established methods. Compared to the shCtrl, tumor lesions in the shCENPF group were scarcely visible in the liver (Fig. 5D). However, upregulating USP4 expression could greatly enhance the the ability of CENPF-knockdown CRC cell to form liver micrometastases (Fig. 5D). The same result was also observed in HE stains result (Fig. 5E). Thus, we proved that CENPF can positively be regulated by USP4 and affects the metastatic ability of CRC cells in vitro and in vivo.

### The USP4-CENPF axis was correlated with clinical outcomes of CRC patients

**Fig. 6:**
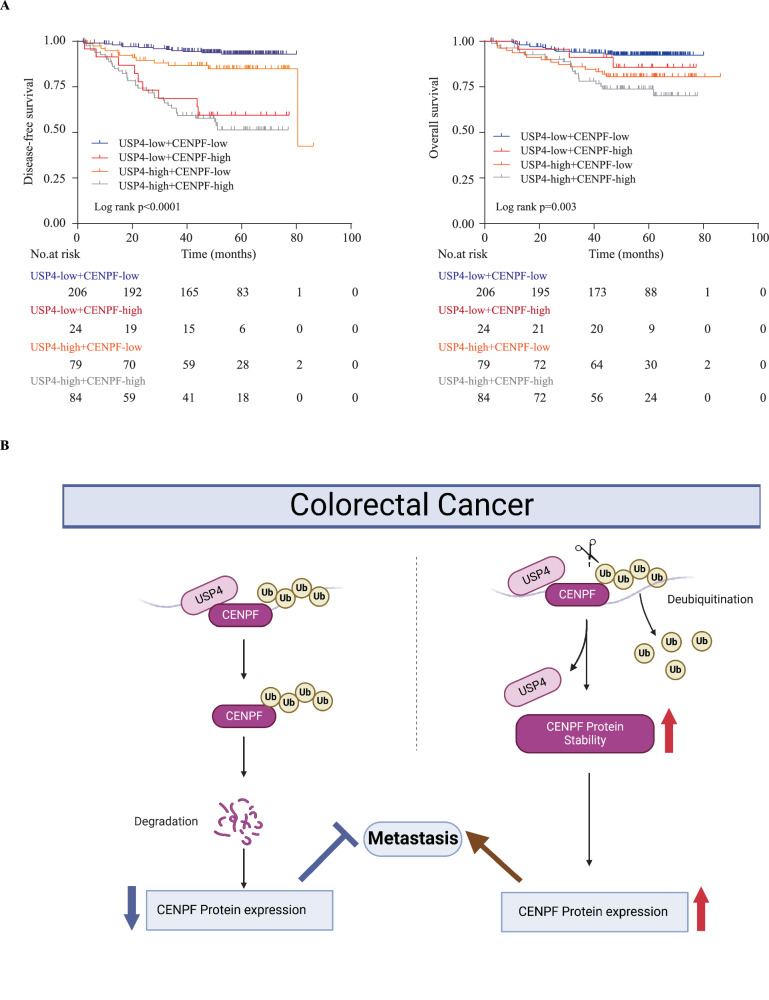
Schematic model illustrating the role of USP4-CENPF axis in mediating CRC metastasis, and this relationship affects CRC patient outcomes. **A** Kaplan–Meier plots illustrating OS and DFS rates of CRC patients, segregated by USP4 and CENPF protein expression levels, in cohort I. Displayed *p*-values indicate significance. **B** A schematic representation detailing the interplay within the USP4-CENPF pathway and its role in curtailing CRC metastasis.

Expanding on previous discoveries linking CENPF and USP4 to CRC patient prognosis and their involvement in CRC metastasis regulation, we explored the prognostic relevance of USP4-CENPF association. We categorized samples from cohort I into four groups according to their USP4 and CENPF protein levels determined by IHC analysis. These categories comprised low USP4/low CENPF, low USP4/high CENPF, high USP4/low CENPF, and high USP4/high CENPF. Subsequently, we conducted comparisons of clinical outcomes among these groups. Kaplan–Meier analysis suggested that patients with high USP4 and high CENPF expression tended to have the poorest DFS and OS compared with the other groups (Fig. 6A). These data imply that the USP4–CENPF axis plays a role in CRC development.

## Discussion

Tumor recurrence is identified as a significant adverse prognostic indicator in patients with colorectal cancer (CRC) following curative resection [39]. In our study, we’re looking into new key genes for CRC using bioinformatics analysis, especially those linked to autophagy-related genes. We firstly identified CENPF as a top candidate gene in CRC progression, examined its functional role in metastatic progression and demonstrated the therapeutic value of targeting CENPF in inhibiting CRC liver metastasis. Mechanically, we found CENPF protein could undergo ubiquitination, leading to subsequent proteasomal degradation. And USP4 as a deubiquitinating enzyme (DUB), interacted with and deubiquitinated CENPF, thereby stabilizing it. Taken together, our data showed that a novel USP4-CENPF axis played an important regulatory role in CRC metastasis and may serve as a potential target for CRC treatment.

Due to the complexity and variability of CRC, effective targeted therapies for CRC and the availability of effective signatures that can accurately predict recurrence remain limited. Autophagy, as a mechanism supporting cell survival, has emerged as a pivotal factor in cancer metastasis. Its impact can be twofold: either promoting or inhibiting metastasis, contingent upon factors such as cancer cell subtype, the tumor microenvironment, and the stage of tumor progression [40, 41]. We conducted correlation analysis using multiple transcriptome sequencing datasets in CRC and an autophagy gene list to identify candidate gene sets. We then intersected the differences between cancer and adjacent tissues to identify potential important targets for CRC treatment, in which is a candidate set comprising 54 genes. Among them, fifteen candidate genes, including DACH1, GALNT6, IFITM1, EGFL6, WNT5A, CDK1, GDF15, SOX9, KIF23, CPNE1, BHLHE40, NEK2, ASPM, PLA2G16, and PMEPA1, have been proven to play important roles in CRC [42–56], which indicated a certain degree of reliability in our screening process. Using two different siRNAs to target candidate genes, we examined their effects on the migratory capacity of HCT116 colorectal cancer cells. Interestingly, knocking down CENPF with siRNA showed the most pronounced inhibition of HCT116 migration, which has not been previously reported. Therefore, the role of CENPF in CRC has become the focus of our research. Moreover, leveraging data from GEO, TCGA databases, and our own colorectal cancer tissue microarrays from two different centers, we explored CENPF expression patterns and prognostic significance in CRC. Consistently, findings indicated upregulation of both CENPF mRNA and protein in colorectal tumors compared to adjacent normal tissues. Similarly, we observed that CRC with recurrence exhibited significantly higher levels of CENPF than CRC without recurrence. In addition, CENPF expression correlated with CRC prognosis, showing more significant predictive efficacy in disease-free survival, indicating its potential oncogenic role in CRC metastasis. Previous results have demonstrated that CENPF was highly expressed in the lung adenocarcinoma (LUAD), and CENPF expression correlated with T stage and poor prognosis [57]. Moreover, CENPF knockout significantly inhibited LUAD cell growth, the tumor growth of mice [57]. Also, CENPF is markedly elevated in pancreatic cancer (PC) and linked to poor patient outcomes [5]. Knocking down CENPF inhibits PC cell proliferation, migration, and EMT, inducing G2/M phase cell cycle arrest and restraining in vivo pancreatic cell growth [5]. Importantly, knocking down CENPF expression significantly altered invasive and migratory capacity of CRC cells, as evidenced from a series of in vitro experiments and in xenograft nude mice models of liver metastasis in vivo. Therefore, the consistent results from our comprehensive study verified that CENPF functions as a novel metastasis-promoting gene in CRC.

Another major finding of our study is that we’ve uncovered the role of ubiquitination in controlling CENPF protein expression and its functions. Until now, there have been few reports confirming the factors that regulate CENPF expression. Previous research has shown that several members of the CENP family, including CENPA, CENPH, and CENPN, are degraded via the ubiquitin-proteasome pathway [15–17]. First, we discovered that MG132 effectively blocked the degradation of CENPF protein and CENPF underwent ubiquitination modifications, suggesting that CENPF could be regulated by ubiquitin-proteasome axis. To further identify the DUBs that can potentially deubiquitinate and stabilize CENPF, we screened a human DUB expression library consisting of 53 DUBs-Flag plasmids in HEK293T cells. The top 18 knockdowns of DUB genes that stabilized CENPF level most significantly were selected for a second-round screening genes. Each of those DUBs was then co-overexpressed together with HA-CENPF in HCT116 cells. Coimmunoprecipitation (co-IP) experiments showed the interaction exists exclusively between USP4 and CENPF. Based on two rounds of screening, we propose that USP4 is the most likely deubiquitinase regulating CENPF stability. Mechanically, USP4 interacts with CENPF and decreases CENPF ubiquitination levels, thus stabilizing it. Accordingly, USP4 expression was significantly positively correlated with CENPF in human CRC samples from two different tertiary hospitals in China, as confirmed by immunohistochemistry. Importantly, the interaction of CENPF and USP4 then controlled the invasion and migration of colorectal cancer. Previous evidence has confirmed a critical role for USP4 in regulating p53, TGFβ, Wnt/β-catenin, and NF-κB signaling, implicating dysregulation of USP4 expression in the development of cancer [58]. In CRC, USP4 has been shown to promote colorectal cancer cell metastasis in vitro and in vivo by regulating the stability and activity of β-catenin and PRL-3 [23, 25]. Previous studies showed that mutating the catalytic residue Cys311 to Ala abolishes USP4’s deubiquitination and stabilization of β-catenin. In our study, the C311S mutation in USP4 also lost its ability to stabilize and deubiquitinate CENPF. Thus, we speculate that CENPF is a key downstream target of USP4 in regulating colorectal cancer metastasis. Furthermore, high USP4 and high CENPF are significant determinants of poor survival in patients with CRC.

This study had some limitations as follows. First, we explained how post-translational modifications regulate CENPF’s abnormal expression and function. However, we also found that CENPF transcription levels are abnormally elevated in colorectal cancer. Understanding the mechanisms behind this upregulation is essential for effectively targeting CENPF abnormalities. Second, the role of the CENPF-USP4 axis in CRC metastasis has been identified, but the specific downstream molecular mechanisms remain unclear and require further investigation, In particular, the role of autophagy activation in mediating CRC metastasis through CENPF requires more in-depth exploration.

In summary, our groundbreaking research, for the first time, has unveiled CENPF as a novel promoter of CRC metastasis and elucidate the molecular mechanism of the interaction between CENPF and USP4 in inducing migration and invasion of colorectal cancer cells, evidenced from molecular, cellular, animal models, and clinical specimens. Thus, our findings provide a theoretical foundation and innovative insights for the potential development of a possible treatment strategy targeting the USP4-CENPF for CRC metastasis.

## Supplementary information

Supplementary Material

## Data Availability

Data and materials supporting the findings of this study are available from the corresponding author (P Chi) upon reasonable request.
