# Supplementary material for: USP4-mediated CENPF deubiquitylation regulated tumor metastasis in colorectal cancer
Source: Cell Death Dis. 2025 Feb 8;16(1):81. doi: 10.1038/s41419-025-07424-3 (PMC11807140; doi:10.1038/s41419-025-07424-3)

**Supplementary Materials**

**USP4-mediated CENPF deubiquitylation regulated tumor metastasis in colorectal cancer**

Zhongdong Xie^1,2†^, Hanbin Lin^3, 4†^, [Yuecheng](https://pubmed.ncbi.nlm.nih.gov/?size=50&term=Chen+Y&cauthor_id=32836062) Wu^4†^, Yanan Yu^5†^, Xintong Liu^6†^, Yating Zheng^2^, Xiaojie Wang^1^, Jiashu Wu^7^, [Meifang Xu](https://pubmed.ncbi.nlm.nih.gov/?term=Xu+M&cauthor_id=36249275)^8^, Yuting Han^4^, [Qiongying Zhang](https://pubmed.ncbi.nlm.nih.gov/?size=50&term=Zhang+Q&cauthor_id=32002035)^9^, Yu Deng^1^, Lin Lin^8^, Linzhu Yan^1^, Qingyun Li^1^, [Xinjian Lin](https://pubmed.ncbi.nlm.nih.gov/?term=Lin+X&cauthor_id=36614238)^4*^, Ying Huang^1*^, Pan Chi^1*^

**Table of Contents:**

Supplemental Table 1

Supplemental Table 2

Supplemental Table 3

Supplemental Table 4

Supplemental Table 5

Supplemental Table 6

Supplemental Table 7

Supplemental Figure 1

Supplemental Figure 2

Supplemental Figure 3

Supplemental Figure 4

Supplemental Figure 5

Supplemental Figure 6

Supplemental Figure 7

Supplemental Figure 8

Supplemental Figure 9

Supplemental Figure 10

Supplemental Figure 11

Supplemental Figure 12

Full and uncropped western blots

**Supplemental Table 1. Clinical characteristics of patient with CRC dichotomized by CENPF protein expression at the cut-off in the two cohorts**

| **Characteristics** | | **Cohort I (n=393)** | | |  | **Cohort II (n=126)** | | | |  |
| --- | --- | --- | --- | --- | --- | --- | --- | --- | --- | --- |
|  |  | **Low-risk group**  **(n=285)** | **High-risk group**  **(n=108)** | **p Value*** |  | **Low-risk group**  **(n=86)** | | **High-risk group**  **(n=40)** | **p Value*** | |
| Sex (n (%) | |  |  | 0.477 | | |  |  | 0.787 |  |
| Female |  | 106 (37.2) | 36 (33.3) |  | | | 28 (32.6) | 14 (35.0) |  |  |
| Male |  | 179 (62.8) | 72 (66.7) |  | | | 58 (67.4) | 26 (65.0) |  |  |
| Age (years) | |  |  | 0.417 | | |  |  | 0.626 |  |
| <60 |  | 113 (39.6) | 38 (35.2) |  | | | 34 (39.5) | 14 (35.0) |  |  |
| ≥60 |  | 172 (60.4) | 70 (64.8) |  | | | 52 (60.5) | 26 (65.0) |  |  |
| Lymphovascular invasion, n (%) | |  |  | 0.316 | | |  |  | 1.000 |  |
| No |  | 251 (88.1) | 91 (84.3) |  | | | 74 (87.2) | 34 (82.5) |  |  |
| Yes |  | 34 (11.9) | 17 (15.7) |  | | | 7 (8.1) | 4 (10.0) |  |  |
| Missing |  | 0 (0.0) | 0 (0.0) |  | | | 5 (4.7) | 2 (7.5) |  |  |
| Perineural invasion, n (%) | |  |  | 0.726 | | |  |  | 1.000 |  |
| No |  | 258 (90.5) | 99 (91.7) |  | | | 78 (90.7) | 39 (97.5) |  |  |
| Yes |  | 27 (9.5) | 9 (8.3) |  | | | 1 (1.2) | 0 (0.0) |  |  |
| Missing |  | 0 (0.0) | 0 (0.0) |  | | | 7 (8.1) | 1 (2.5) |  |  |
| Adjuvant chemotherapy (n (%)) | |  |  | 0.069 | | |  |  | 0.393 |  |
| No |  | 121 (42.5) | 35 (32.4) |  | | | 35 (40.7) | 16 (40.0) |  |  |
| Yes |  | 164 (57.5) | 73 (67.6) |  | | | 51 (59.3) | 24 (60.0) |  |  |
| Missing |  | 0 (0.0) | 0 (0.0) |  | | | 0 (0.0) | 0 (0.0) |  |  |
| Serum CEA, n (%) | |  |  | 0.392 | | |  |  | 0.740 |  |
| <5 ng/mL |  | 167 (58.6) | 67 (62.0) |  | | | 50 (58.1) | 22 (55.0) |  |  |
| ≥5 ng/mL |  | 106 (37.2) | 41 (38.0) |  | | | 36 (41.9) | 18 (45.0) |  |  |
| Missing |  | 12 (4.2) | 0 (0.0) |  | | | 0 (0.0) | 0 (0.0) |  |  |
| Serum CA19-9, n (%) | |  |  | 0.358 | | |  |  | 0.435 |  |
| <37 U/mL |  | 232 (81.4) | 96 (88.9) |  | | | 70 (81.4) | 31 (77.5) |  |  |
| ≥37 U/mL |  | 40 (14.0) | 12 (11.1) |  | | | 14 (16.3) | 9 (22.5) |  |  |
| Missing |  | 13 (4.6) | 0 (0.0) |  | | | 2 (2.3) | 0 (0.0) |  |  |
| Tumor size, n (%) | |  |  | 0.164 | | |  |  | 0.603 |  |
| <4 cm |  | 160 (56.1) | 69 (63.9) |  | | | 22 (25.6) | 12 (30.0) |  |  |
| ≥4 cm |  | 125 (43.9) | 39 (36.1) |  | | | 64 (74.4) | 28 (70.0) |  |  |
| Missing |  | 0 (0.0) | 0 (0.0) |  | | | 0 (0.0) | 0 (0.0) |  |  |
| Differentiation grade (n (%)) | |  |  | 0.572‡ | | |  |  | 0.699‡ |  |
| Well |  | 26 (9.1) | 10 (9.3) |  | | | 8 (9.3) | 3 (7.5) |  |  |
| Moderately |  | 217 (76.1) | 79 (73.1) |  | | | 54 (62.8) | 28 (70.0) |  |  |
| Poorly |  | 41 (14.4) | 19 (17.6) |  | | | 24 (27.9) | 9 (22.5) |  |  |
| Missing |  | 0 (0.0) | 0(0.0) |  | | | 0 (0.0) | 0 (0.0) |  |  |
| TNM stage (n (%)) | |  |  | 0.002‡ | | |  |  | 0.750‡ |  |
| I |  | 69 (24.2) | 17 (15.7) |  | | | 7 (8.1) | 4 (10.0) |  |  |
| II |  | 121 (42.5) | 36 (33.3) |  | | | 38 (44.2) | 17 (35.0) |  |  |
| III |  | 71 (24.9) | 39 (36.1) |  | | | 35 (40.7) | 17 (35.0) |  |  |
| IV |  | 24 (8.4) | 16 (14.8) |  | | | 6 (7.0) | 2 (7.5) |  |  |
| Tumor location, n (%) | |  |  | 0.565‡ | | |  |  | 0.791‡ |  |
| Right | | 50 (17.5) | 15 (13.9) |  | | | 35 (40.7) | 16 (40.0) |  |  |
| Left | | 108 (37.9) | 43 (39.8) |  | | | 39 (45.3) | 17 (42.5) |  |  |
| Rectum | | 127 (44.6) | 50 (46.3) |  | | | 12 (14.0) | 7 (17.5) |  |  |

*χ2 test or Fisher’s exact test.

‡Mann–Whitney U test (non-parametric). Missing values are excluded for all statistic tests.

CA19-9, carbohydrate antigen 19-9; CEA, carcinoembryonic antigen; TNM, tumor-node-metastasis.

**Supplemental Table 2. Inter-observer agreement between 2 observers in the scoring of CENPF immunostaining using Cohen^’^ s Kappa index**

| CENPF score of individual tissue cores | | Observer #1 | | | |
| --- | --- | --- | --- | --- | --- |
|  |  | Score 1 | Score 2 | Score 3 | Score 4 |
| Observer #2 | Score 1 | **133** | 20 | 0 | 0 |
|  | Score 2 | 1 | **100** | 1 | 2 |
|  | Score 3 | 4 | 0 | **94** | 0 |
|  | Score 4 | 0 | 12 | 4 | **38** |

Kappa with linear weighting=0.900, p<0.001.

**Supplemental Table 3. Inter-observer agreement between 2 observers in the scoring of USP4 immunostaining using Cohen^’^ s Kappa index**

| USP4 score of individual tissue cores | | Observer #1 | | | |
| --- | --- | --- | --- | --- | --- |
|  |  | Score 1 | Score 2 | Score 3 | Score 4 |
| Observer #2 | Score 1 | **189** | 11 | 2 | 0 |
|  | Score 2 | 9 | **99** | 2 | 0 |
|  | Score 3 | 1 | 2 | **63** | 3 |
|  | Score 4 | 0 | 1 | 4 | **7** |

Kappa with linear weighting=0.858, p<0.001.

**Supplemental Table 4. Autophagy gene list**

| AMBRA1 | CALCOCO2 | EIF2AK2 | HGS | MAPK9 | RAB33B | WDR45L |
| --- | --- | --- | --- | --- | --- | --- |
| APOL1 | CAMKK2 | EIF2AK3 | HIF1A | MBTPS2 | RAB5A | WIPI1 |
| ARNT | CANX | EIF2S1 | HSP90AB1 | MLST8 | RAB7A | WIPI2 |
| ARSA | CAPN1 | EIF4EBP1 | HSPA5 | MTMR14 | RAC1 | ZFYVE1 |
| ARSB | CAPN10 | EIF4G1 | HSPA8 | MTOR | RAF1 |  |
| ATF4 | CAPN2 | ERBB2 | HSPB8 | MYC | RB1 |  |
| ATF6 | CAPNS1 | ERN1 | IFNG | NAF1 | RB1CC1 |  |
| ATG10 | CASP1 | ERO1L | IKBKB | NAMPT | RELA |  |
| ATG12 | CASP3 | FADD | IKBKE | NBR1 | RGS19 |  |
| ATG16L1 | CASP4 | FAM48A | IL24 | NCKAP1 | RHEB |  |
| ATG16L2 | CASP8 | FAS | IRGM | NFE2L2 | RPS6KB1 |  |
| ATG2A | CCL2 | FKBP1A | ITGA3 | NFKB1 | RPTOR |  |
| ATG2B | CCR2 | FKBP1B | ITGA6 | NKX2-3 | SAR1A |  |
| ATG3 | CD46 | FOS | ITGB1 | NLRC4 | SERPINA1 |  |
| ATG4A | CDKN1A | FOXO1 | ITGB4 | NPC1 | SESN2 |  |
| ATG4B | CDKN1B | FOXO3 | ITPR1 | NRG1 | SH3GLB1 |  |
| ATG4C | CDKN2A | GAA | KIAA0226 | NRG2 | SIRT1 |  |
| ATG4D | CFLAR | GAA | KIAA0652 | NRG3 | SIRT2 |  |
| ATG5 | CHMP2B | GABARAP | KIAA0831 | P4HB | SPHK1 |  |
| ATG7 | CHMP4B | GABARAP | KIF5B | PARK2 | SPNS1 |  |
| ATG9A | CLN3 | GABARAPL1 | KLHL24 | PARP1 | SQSTM1 |  |
| ATG9B | CTSB | GABARAPL1 | LAMP1 | PEA15 | ST13 |  |
| ATIC | CTSD | GABARAPL2 | LAMP2 | PELP1 | STK11 |  |
| BAG1 | CTSL1 | GABARAPL2 | MAP1LC3A | PEX14 | TBK1 |  |
| BAG3 | CX3CL1 | GAPDH | MAP1LC3B | PEX3 | TM9SF1 |  |
| BAK1 | CXCR4 | GAPDH | MAP1LC3C | PIK3C3 | TMEM49 |  |
| BAX | DAPK1 | GNAI3 | MAP2K7 | PIK3R4 | TMEM74 |  |
| BCL2 | DAPK2 | GNAI3 | MAPK1 | PINK1 | TNFSF10 |  |
| BCL2L1 | DDIT3 | GNB2L1 | MAPK3 | PPP1R15A | TP53 |  |
| BECN1 | DIRAS3 | GNB2L1 | MAPK8 | PRKAB1 | TP53INP2 |  |
| BID | DLC1 | GOPC | MAPK8IP1 | PRKAR1A | TP63 |  |
| BIRC5 | DNAJB1 | GOPC | USP10 | PRKCD | TP73 |  |
| BIRC6 | DNAJB9 | GRID1 | UVRAG | PRKCQ | TSC1 |  |
| BNIP1 | DRAM1 | GRID1 | VAMP3 | PTEN | TSC2 |  |
| BNIP3 | EDEM1 | GRID2 | VAMP7 | PTK6 | TUSC1 |  |
| BNIP3L | EEF2 | GRID2 | VEGFA | RAB11A | ULK1 |  |
| C12orf44 | EEF2K | HDAC1 | WDFY3 | RAB1A | ULK2 |  |
| C17orf88 | EGFR | HDAC6 | WDR45 | RAB24 | ULK3 |  |

**Supplemental Table 5. Sequences of siRNA and shRNA for this study**

|  | **siRNA duplexs :5’ to 3’** | | Concentration |
| --- | --- | --- | --- |
| USP4-siRNA1 | | GGCUCUGGAACAAAUACAUTT (sense)  AUGUAUUUGUUCCAGAGCCTT (antisense) | 20nM |
| USP4-siRNA2 | | CUGCAUAUGCGAAGAACAATT (sense)  UUGUUCUUCGCAUAUGCAGTT (antisense) | 20nM |
| USP4-siRNA3 | | CCCAACUGUAAGAAGCAUCAATT (sense)  UUGAUGCUUCUUACAGUUGGGTT (antisense) | 20nM |
| S100A2-siRNA1 | | GGGAGAAAGUGGAUGAGGATT (sense) | 20nM |
|  |  | UCCUCAUCCACUUUCUCCCTT (antisense) |  |
| S100A2-siRNA2 | | UGAAGGAACUUCUGCACAATT (sense) | 20nM |
|  |  | UUGUGCAGAAGUUCCUUCATT (antisense) |  |
| TMEM158-siRNA1 | | CCUCCAAUGCUUCAGUCAATT (sense) | 20nM |
|  |  | UUGACUGAAGCAUUGGAGGTT (antisense) |  |
| TMEM158-siRNA2 | | GCUGCAUUUCUGCUGCCUATT (sense) | 20nM |
|  |  | UAGGCAGCAGAAAUGCAGCTT (antisense) |  |
| GPR143-siRNA1 | | GGUUGUCGAAUAUCAUCAATT (sense) | 20nM |
|  |  | UUGAUGAUAUUCGACAACCTT (antisense) |  |
| GPR143-siRNA2 | | GCUAUGCAGUGGAUGCUUATT (sense) | 20nM |
|  |  | UAAGCAUCCACUGCAUAGCTT (antisense) |  |
| RNF43-siRNA1 | | GUACCAGCAGUCUGUUCAATT (sense) | 20nM |
|  |  | UUGAACAGACUGCUGGUACTT (antisense) |  |
| RNF43-siRNA2 | | GCAUGUUCAACAUCACAGATT (sense) | 20nM |
|  |  | UCUGUGAUGUUGAACAUGCTT (antisense) |  |
| CENPF-siRNA1 | | GCAGCGAGAUUGUUCUCAATT (sense) | 20nM |
|  |  | UUGAGAACAAUCUCGCUGCTT (antisense) |  |
| CENPF-siRNA2 | | GGCAGAGAUUCAAGAAUUATT (sense) | 20nM |
|  |  | UAAUUCUUGAAUCUCUGCCTT (antisense) |  |
| LZTS3-siRNA1 | | GCGAGAGCGACGAGGCUAATT (sense) | 20nM |
|  |  | UUAGCCUCGUCGCUCUCGCTT (antisense) |  |
| LZTS3-siRNA2 | | GCUAGACCAGUGCUCAGAATT (sense) | 20nM |
|  |  | UUCUGAGCACUGGUCUAGCTT (antisense) |  |
| TMEM97-siRNA1 | | GGUAUGCUAAGGAGUUCAATT (sense) | 20nM |
|  |  | UUGAACUCCUUAGCAUACCTT (antisense) |  |
| TMEM97-siRNA2 | | GACUUUGCAUGAACGGUUATT (sense) | 20nM |
|  |  | UAACCGUUCAUGCAAAGUCTT (antisense) |  |
| AHCY-siRNA1 | | GCAAGUUUGACAACCUCUATT (sense) | 20nM |
|  |  | UAGAGGUUGUCAAACUUGCTT (antisense) |  |
| AHCY-siRNA2 | | CCAGCUUCGUGAUGAGUAATT (sense) | 20nM |
|  |  | UUACUCAUCACGAAGCUGGTT (antisense) |  |
| RHBDF2-siRNA1 | | GUGAGUUCAUGCACGGCUATT (sense) | 20nM |
|  |  | UAGCCGUGCAUGAACUCACTT (antisense) |  |
| RHBDF2-siRNA2 | | GGAACAAAGGUGUGUACGATT (sense) | 20nM |
|  |  | UCGUACACACCUUUGUUCCTT (antisense) |  |
| SERPINH1-siRNA1 | | GCAGCAAGCAGCACUACAATT (sense) | 20nM |
|  |  | UUGUAGUGCUGCUUGCUGCTT (antisense) |  |
| SERPINH1-siRNA2 | | GGACAGGCCUCUACAACUATT (sense) | 20nM |
|  |  | UAGUUGUAGAGGCCUGUCCTT (antisense) |  |
| COL1A1-siRNA1 | | CCAUCAAAGUCUUCUGCAATT (sense) | 20nM |
|  |  | UUGCAGAAGACUUUGAUGGTT (antisense) |  |
| COL1A1-siRNA2 | | GCGAGGAGCUCGAGGUGAATT (sense) | 20nM |
|  |  | UUCACCUCGAGCUCCUCGCTT (antisense) |  |
| RRP9-siRNA1 | | GGACUGUACGUGUGUGGAATT (sense) | 20nM |
|  |  | UUCCACACACGUACAGUCCTT (antisense) |  |
| RRP9-siRNA2 | | GGACGGAAGCUGCAUGUGATT (sense) | 20nM |
|  |  | UCACAUGCAGCUUCCGUCCTT (antisense) |  |
| KIF20A-siRNA1 | | GCAUGAUUGUCAAUGUGAATT (sense) | 20nM |
|  |  | UUCACAUUGACAAUCAUGCTT (antisense) |  |
| KIF20A-siRNA2 | | CGAACUGCUUUAUGACCUATT (sense) | 20nM |
|  |  | UAGGUCAUAAAGCAGUUCGTT (antisense) |  |
| SLC5A6-siRNA1 | | GGUUCUAUUCCUUGUCUUATT (sense) | 20nM |
|  |  | UAAGACAAGGAAUAGAACCTT (antisense) |  |
| SLC5A6-siRNA2 | | GGCAACUGUUACGAUGGAATT (sense) | 20nM |
|  |  | UUCCAUCGUAACAGUUGCCTT (antisense) |  |
| BGN-siRNA1 | | GGAAGCUGCAGAAGCUCUATT (sense) | 20nM |
|  | | UAGAGCUUCUGCAGCUUCCTT (antisense) |  |
| BGN-siRNA2 | | GCAUCAGCCUCUUCAACAATT (sense) | 20nM |
|  | | UUGUUGAAGAGGCUGAUGCTT (antisense) |  |
| GPSM2-siRNA1 | | GAGAUGUUGCUUACAAGAATT (sense) | 20nM |
|  |  | UUCUUGUAAGCAACAUCUCTT (antisense) |  |
| GPSM2-siRNA2 | | GGUCUGAGCUACAGCACAATT (sense) | 20nM |
|  |  | UUGUGCUGUAGCUCAGACCTT (antisense) |  |
| SLC6A6-siRNA1 | | GAGAAGUGGUCUAGCAAGATT (sense) | 20nM |
|  |  | UCUUGCUAGACCACUUCUCTT (antisense) |  |
| SLC6A6-siRNA2 | | CGGCUAUGCCUCCGUUGUATT (sense) | 20nM |
|  |  | UACAACGGAGGCAUAGCCGTT (antisense) |  |
| HAUS6-siRNA1 | | GGAAGAAGUUGGUCAUCUATT (sense) | 20nM |
|  |  | UAGAUGACCAACUUCUUCCTT (antisense) |  |
| HAUS6-siRNA2 | | GCAAGAUUUGGAAUGUUUATT (sense) | 20nM |
|  |  | UAAACAUUCCAAAUCUUGCTT (antisense) |  |
| TNFRSF12A-  siRNA1 | | CGCAGGAGAGAGAAGUUCATT (sense) | 20nM |
|  |  | UGAACUUCUCUCUCCUGCGTT (antisense) |  |
| TNFRSF12A-siRNA2 | | GGAGCGCGGACCUGGACAATT (sense) | 20nM |
|  |  | UUGUCCAGGUCCGCGCUCCTT (antisense) |  |
| TCFL5-siRNA1 | | GAAUGUUCCUCUUCAGCAATT (sense) | 20nM |
|  |  | UUGCUGAAGAGGAACAUUCTT (antisense) |  |
| TCFL5-siRNA2 | | CGUUAGGUAGUAGAAACAATT (sense) | 20nM |
|  |  | UUGUUUCUACUACCUAACGTT (antisense) |  |
| SLC7A5-siRNA1 | | GGAAGGGUGAUGUGUCCAATT (sense) | 20nM |
|  |  | UUGGACACAUCACCCUUCCTT (antisense) |  |
| SLC7A5-siRNA2 | | GUGUGAUGACGCUGCUCUATT (sense) | 20nM |
|  |  | UAGAGCAGCGUCAUCACACTT (antisense) |  |
| LY6E-siRNA1 | | GCUCCGACCAGGACAACUATT (sense) | 20nM |
|  | | UAGUUGUCCUGGUCGGAGCTT (antisense) |  |
| LY6E-siRNA2 | | GCUUGAACCAGAAGAGCAATT (sense) | 20nM |
|  | | UUGCUCUUCUGGUUCAAGCTT (antisense) |  |
| SLC12A2-siRNA1 | | AGAUGUUUGCUAAAGGUUATT (sense) | 20nM |
|  |  | UAACCUUUAGCAAACAUCUTT (antisense) |  |
| SLC12A2-siRNA2 | | GGAUAUGUAUAUAAACUUATT (sense) | 20nM |
|  |  | UAAGUUUAUAUACAUAUCCTT (antisense) |  |
| ARNTL2-siRNA1 | | GGAACAGUAAGAAAGACAATT (sense) | 20nM |
|  |  | UUGUCUUUCUUACUGUUCCTT (antisense) |  |
| ARNTL2-siRNA2 | | GGAUGCUUACCCAACUCAATT (sense) | 20nM |
|  |  | UUGAGUUGGGUAAGCAUCCTT (antisense) |  |
| Control siRNA | | UUCUCCGAACGUGUCACGUTT (sense) | 20nM |
|  | | ACGUGACACGUUCGGAGAATT (antisense) |  |
|  | | **shRNA :5’ to 3’** |  |
| CENPF-shRNA1 | | GCGCAGAAUCAAGAGCUAA |  |
| CENPF-shRNA2 | | GCGGGAGAAUGAUUCACUU |  |

**Supplemental Table 6. Sequences of qPCR primers for this study**

|  | **Primers: 5’-3’** | **PCR program** |
| --- | --- | --- |
| USP4 | AGGTGTATTTGCTGGAACTGAAG (forward) | Denatured 30s at 95°C, and followed by 40 cycles of 95°C for 10s, 60°C for 30s |
|  | CTTGCTCAACTGCTCGTAGG (reverse) |  |
| CENPF | TTCAAGAACTCCAACTCCAACTG (forward) |  |
|  | CCTGGTCGTATTGTGCTGTT (reverse) |  |
| GAPDH | GGTGTGAACCATGAGAAGTATGA (forward)  GAGTCCTTCCACGATACCAAAG (reverse) |  |
| S100A2 | CCAGCTTTGTGGGGGAGAAAGT (forward) |  |
|  | TGACAGTGATGAGTGCCAGGAA (reverse) |  |
| TMEM158 | GCATTTCTGCTGCCTAGACTTC (forward) |  |
|  | CTCCACACCACGATGACCAG (reverse) |  |
| GPR143 | CTTCTACGGCTGGACAGGAT (forward) |  |
|  | ACCCACTTGAGACACCTTCC (reverse) |  |
| RNF43 | TGGGGTAATGACGCTGAGAAG (forward) |  |
|  | GTGCCCACCACTGTCATTAGG (reverse) |  |
| LZTS3 | CTCCACCAGCCACATTAACC (forward) |  |
|  | TAGATGACGACGACCCACTC (reverse) |  |
| TMEM97 | CCTGCTGAAGTGGTATGCTAAG (forward) |  |
|  | TGGTGTGAACAGAGTAGATGATTG (reverse) |  |
| AHCY | AACATGATTCTGGACGACGG (forward) |  |
|  | ACTTGCTCTTGGTGACGGAG (reverse) |  |
| RHBDF2 | TGTGTACGAGAGCGTGAAGT (forward) |  |
|  | GTGGTCATTCTGGACACAGC (reverse) |  |
| SERPINH1 | CGACCACCCCTTCATCTTCC (forward) |  |
|  | ACTCGTCTCGCATCTTGTCA (reverse) |  |
| COL1A1 | GAGAGCATGACCGATGGATTC (forward) |  |
|  | CTTCTTGAGGTTGCCAGTCTG (reverse) |  |
| RRP9 | GCTTGACCTTCTCTGTGACAT (forward) |  |
|  | GCCTCTTTGATTCTCCACCAT (reverse) |  |
| KIF20A | AGTCACAGCATCTTCTCAATCAG (forward) |  |
|  | TTCAACCGTTCACCACTCTTC (reverse) |  |
| SLC5A6 | GTGGGCCTGATTGTCAGTCT (forward) |  |
|  | CACACCATTCCTCGGCTTCT (reverse) |  |
| BGN | TGGCCTGAAGCTCAACTACC (forward) |  |
|  | CAGGTCCTCCAGTTCGATGG (reverse) |  |
| GPSM2 | CATCATCTGTTCCTGTGGTATCC (forward) |  |
|  | AGCCTCTTTGTTGTCATTAGTCAT (reverse) |  |
| SLC6A6 | GCTACAAGAATGGTGGAGGTG (forward) |  |
|  | GACACAATTACAACGGAGGCATA (reverse) |  |
| HAUS6 | CGGAAGAAGTTGGTCATCTAAGTT (forward) |  |
|  | GTCTGATTCTGGAGTAGTCTGTCT (reverse) |  |
| TNFRSF12A | GAGAGAAGTTCACCACCCCCAT (forward) |  |
|  | ATGAATGAATGATGAGTGGGCG (reverse) |  |
| TCFL5 | CTGCGGCTACAACTACTGCTT (forward) |  |
|  | GGCAATCCAATATCCTGGTGC (reverse) |  |
| SLC7A5 | GGAACATTGTGCTGGCATTATAC (forward) |  |
|  | TGGACAGGGTGGTGAAGTAG (reverse) |  |
| LY6E | AGGACAACTACTGCGTGACTG (forward) |  |
|  | CCACACCAACATTGACGCCT (reverse) |  |
| SLC12A2 | CAGTCCTTGTTCCTATGGCCT (forward) |  |
|  | AGCTGGGTAGATGTTGTCCT (reverse) |  |
| ARNTL2 | AATCTCCACGCTGGAAGGAC (forward) |  |
|  | TTGGAGGCCAGCTTCTCAAG (reverse) |  |

**Supplemental Table 7. Sequences of primers for molecular cloning**

|  | | **Primers: 5’-3’** |  |
| --- | --- | --- | --- |
| USP4 | AGAGAATTCGGATCC ATGGCGGAAGGTGGAGGC (forward) | |  |
|  | TGGCTCGAGCCCGGG GTTGGTGTCCATGCTGCAAG (reverse) | |  |
| USP4 C311S | AACACCTCCTTCATGAACTCCGCTTTGC (forward) | |  |
|  | CATGAAGGAGGTGTTTCCCAGGTTTCCAA (reverse) | |  |
| USP4-∆DUSP | AGAGAATTCGGATCCATGCCGGATGCGGAGACTCAG | |  |
| USP4-∆UBL1 | GAACTGAAGCAGACCTTGCAGTCAAAATCAA (forward)  GGTCTGCTTCAGTTCCAGCAAATACACCTCG (reverse) | |  |
| USP4-∆USP | CCTGGGCTCGATGATGAATTTTATAAGACACCT (forward)  ATCATCGAGCCCAGGTTGTATATGAGAGGATG (reverse) | |  |
| USP4-∆UBL2 | CCACTGCCCGTGGATGGCTCGGAATGTG (forward)  ATCCACGGGCAGTGGCAGCGTTAGATAGCA (reverse) | |  |


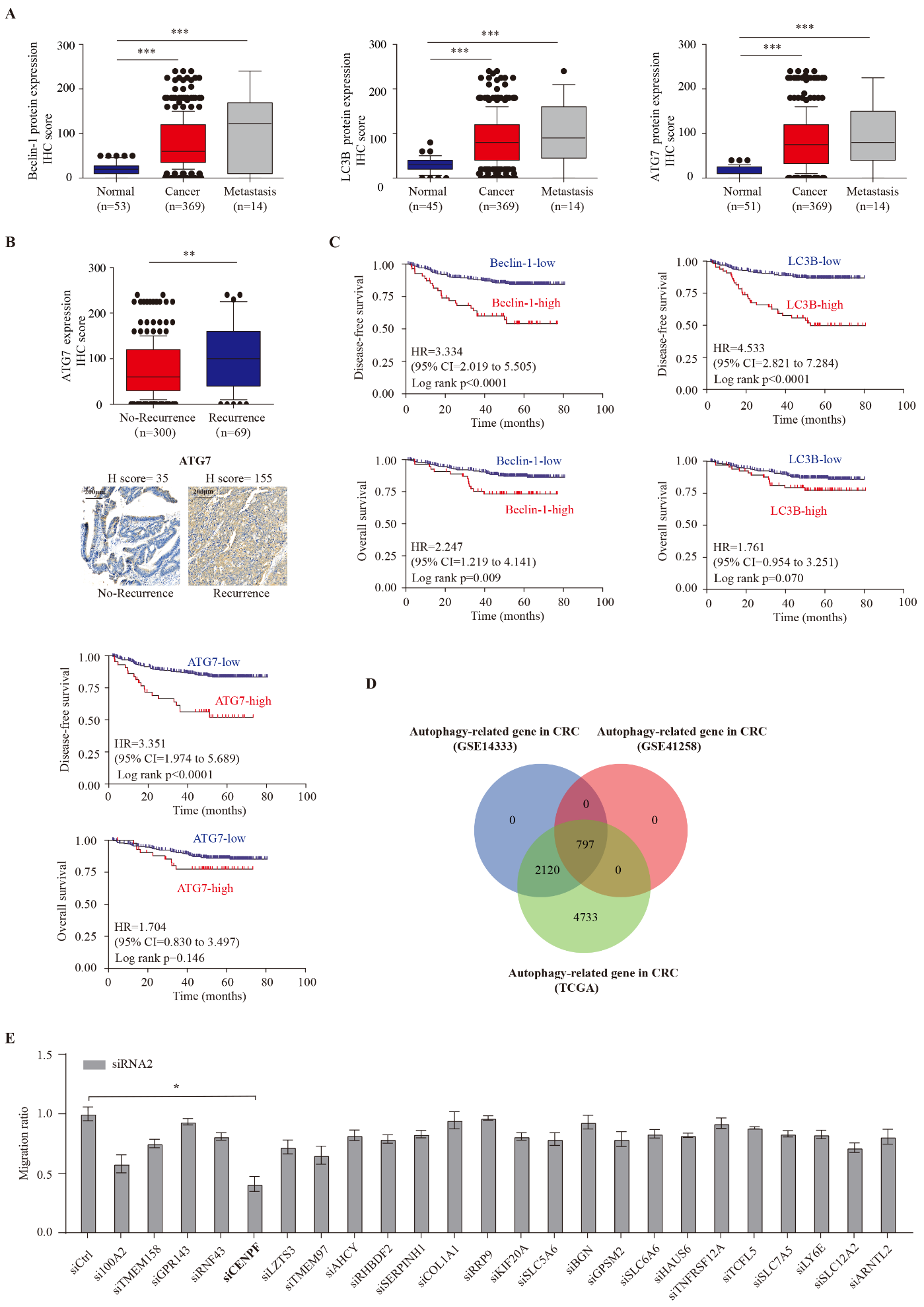
**Supplemental Figure 1. Expression status of key autophagy regulators in colorectal tissues.**

**(A)** IHC staining showing Beclin-1, LC3B, and ATG7 protein levels in cancer and cancer-related specimens from Cohort I. Analyzed using one-way ANOVA and LSD’s test, with significance noted as ***P < 0.001. **(B)** Significant upregulation of ATG7 protein expression in CRC with recurrence compared to CRC without recurrence. Evaluation based on the nonparametric Mann-Whitney test. Significance indicated by **P < 0.01. **(C)** Kaplan–Meier survival curves for disease-free survival (DFS) and overall survival (OS) in CRC patients based on Beclin-1, LC3B, and ATG7 expression levels in Cohort I. Log-rank p-values and Hazard Ratios (HRs) are indicated. **(D)** Venn diagram showing the intersection of autophagy-related genes from the GSE14333, GSE41258, and TCGA datasets. **(E)** HCT116 cells were treated with siRNA2 targeting candidate genes, and transwell assays were performed to assess cell migration. Migration rates are presented as the ratio of each treated group to the control, with bar graphs depicting migration rates for each group. Results were analyzed using the Student’s t-test. *p<0.05.


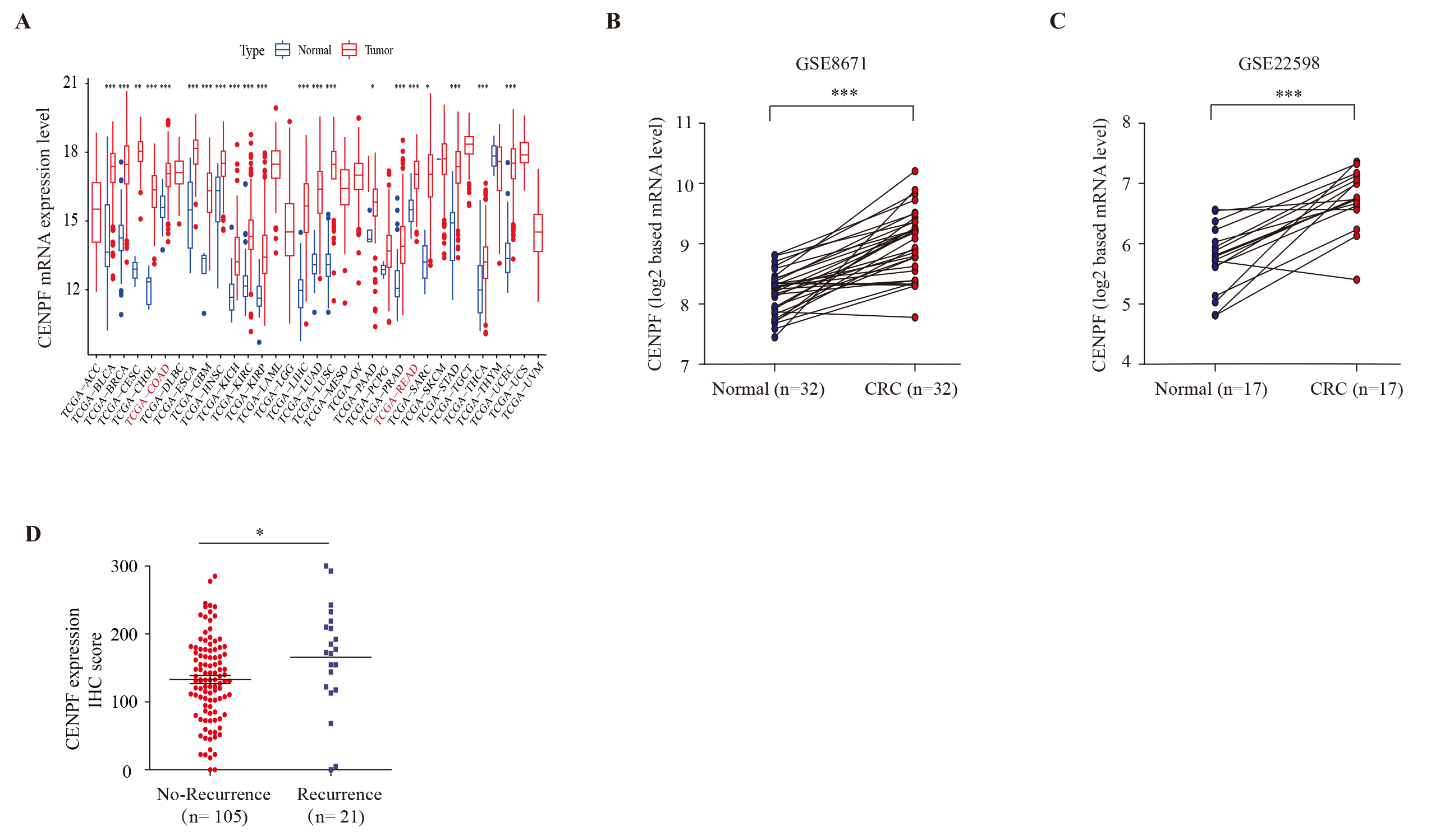


**Supplemental Figure 2. Expression of CENPF was frequently elevated in CRC. (A)** Explore the expression pattern of CENPF mRNA by obtaining data from the TCGA public database for various tumors. Compare the expression of CENPF mRNA between different tumor tissues and adjacent normal tissues using box plots. The blue box indicates normal tissue, while the red box indicates tumor tissue. *p < 0.05, **p < 0.01, and ***p < 0.001. **(B-C)** Bioinformatics analyses of CENPF mRNA expression between cancer and cancer-related specimens in two datasets downloaded from GEO database. "Normal" refers to normal tissue, while "CRC" refers to colorectal cancer tissue. ***p<0.001. **(D)** Immunohistochemistry analysis of CENPF protein levels in CRC with recurrence versus without recurrence in Cohort II. The Mann-Whitney non-parametric test was used to compare the expression differences. *p<0.05.


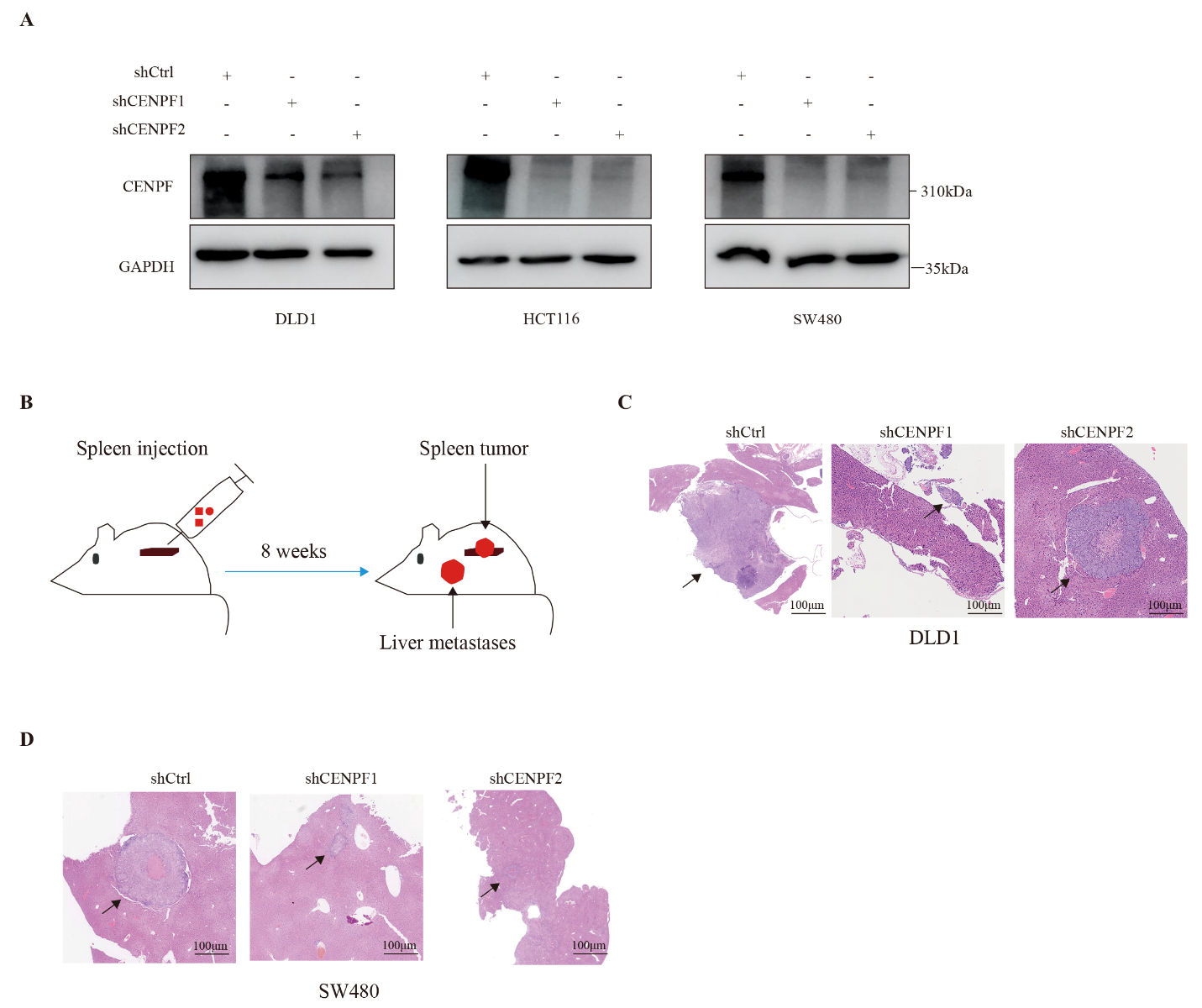


**Supplemental Figure 3.** **Knockdown of CENPF inhibits the ability of colorectal cancer cell to develop liver micrometastases. (A)** The expression level of CENPF in DLD1, HCT116, SW480 cells after transfected with CENPF shRNAs or shCtrl was detected by western blot. Thus, CENPF-knock down stable cell lines were established. **(B)** Schematic diagram of spleen inoculated with CRC cell liver metastasis model in Nude mouse. **(C-D)** Representative HE staining images of DLD1 and SW480 tumors in the 8 weeks after spleen injection. Scale bars, 200 μm.


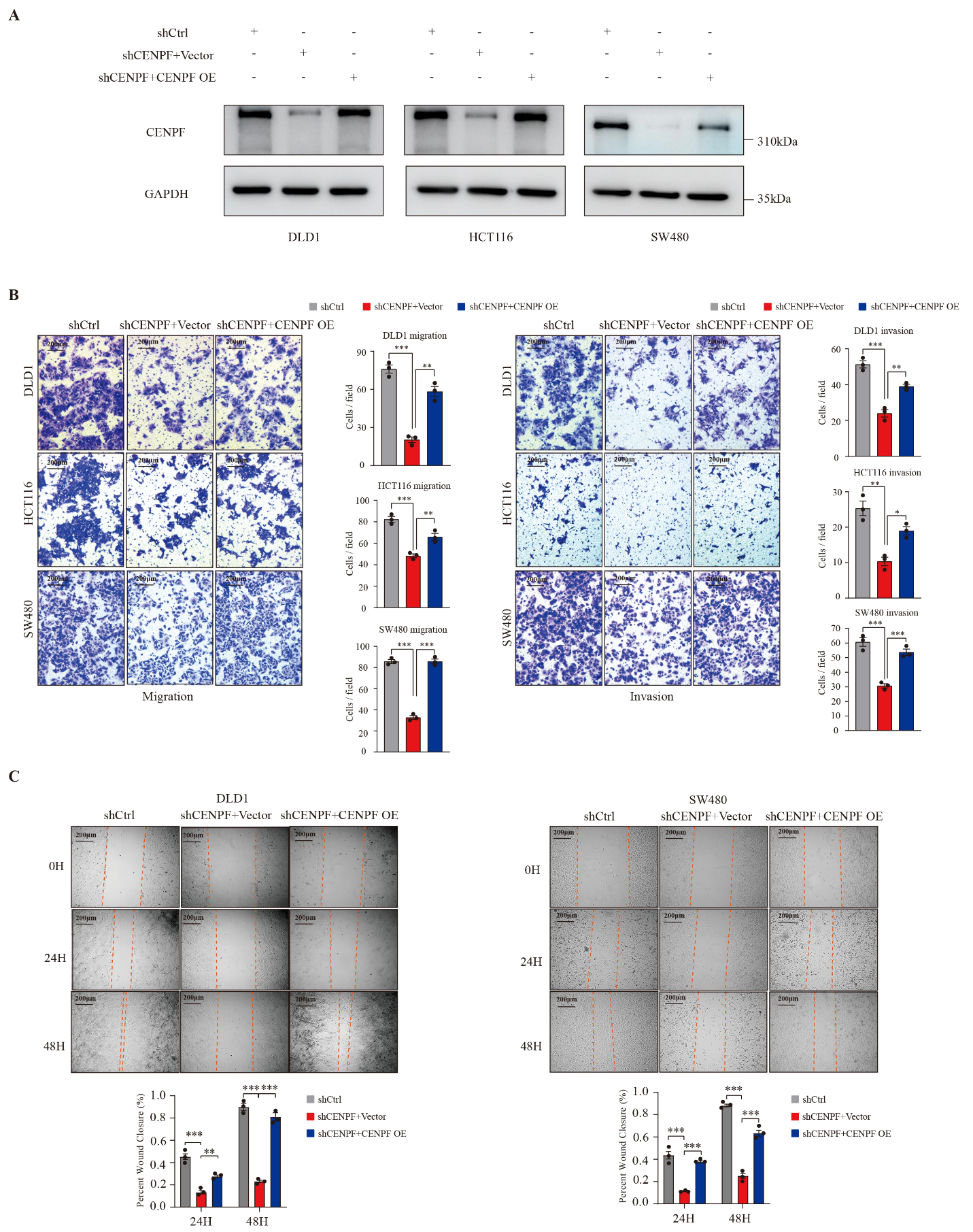


**Supplemental Figure 4. Overexpression of CENPF restores weakened migration and invasion abilities mediated by CENPF knockdown in colorectal cancer cells.**

1. Western blotting results showing the expression of CENPF from samples of the mentioned cells. **(B)** Transwell assays determined the migration rates of specific cells. Data is represented as the average value ± SEM from three distinct tests. Analyzed via One-way ANOVA. ***p<0.001. **(C)** Quantification of the wound healing assays using indicated cells. Differences in the area of wound closure among different groups were compared using one-way ANOVA. **p<0.01, ***p<0.001.


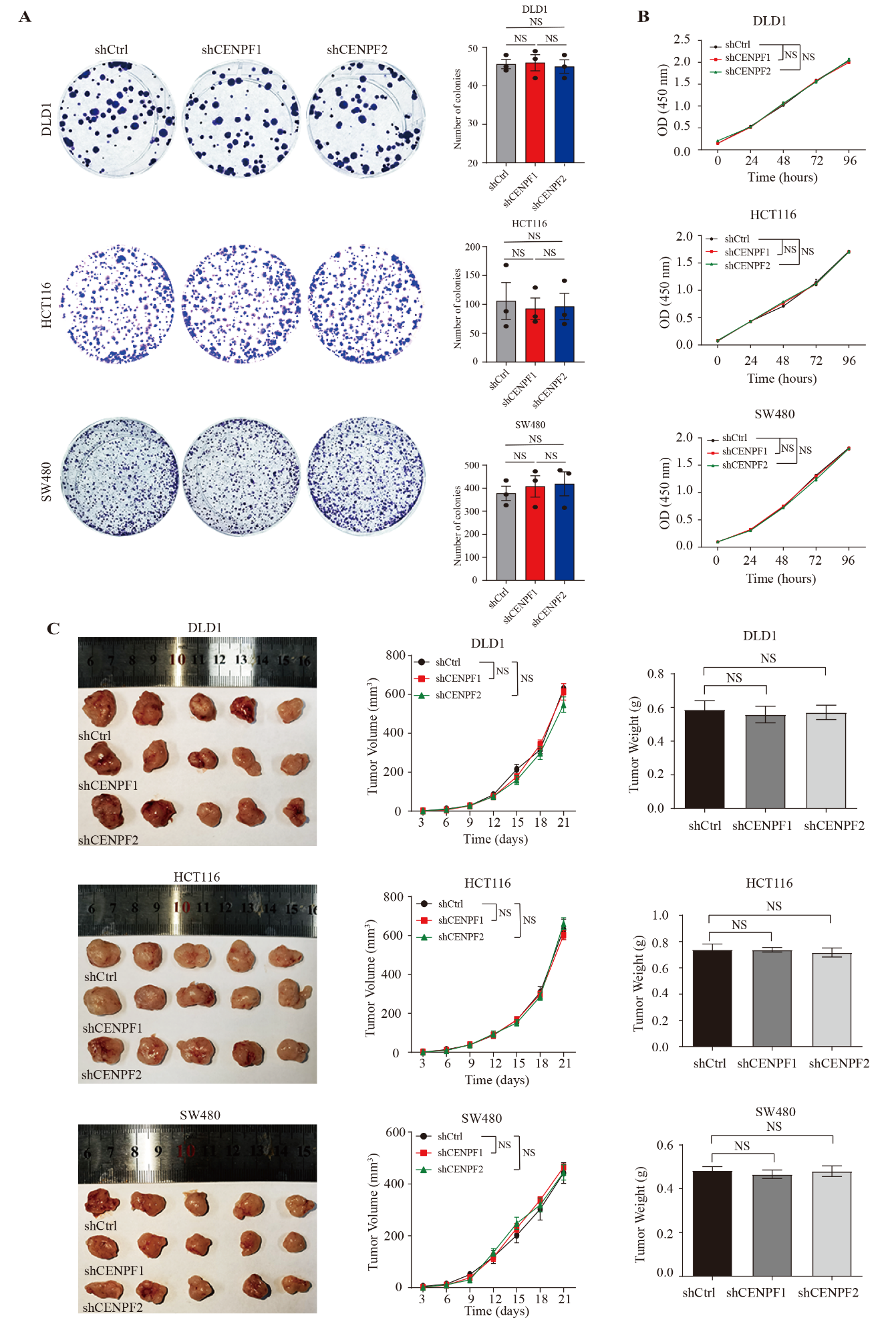


**Supplemental Figure 5. Knockdown of CENPF does not affect CRC cell growth. (A)** Examination of CENPF knockdown's influence on the colony-forming abilities of CRC cells. Results were analyzed using the Student’s t-test. NS. indicates non-significant. **(B)** Proliferation rates of specified cells assessed using the CCK8 assay. Results are denoted as mean ± SEM from three distinct experiments. Assessed by Two-way ANOVA. NS. indicates non-significant. **(C)** Exploration of the influence of CENPF knockdown in HCT116, DLD1, SW480 cells using a xenograft model. Displayed are images of the tumors from mice and a quantification of tumor growth and size. Data represents mean ± SEM for eight subjects per group. Evaluated by Two-way ANOVA. NS. indicates non-significant.


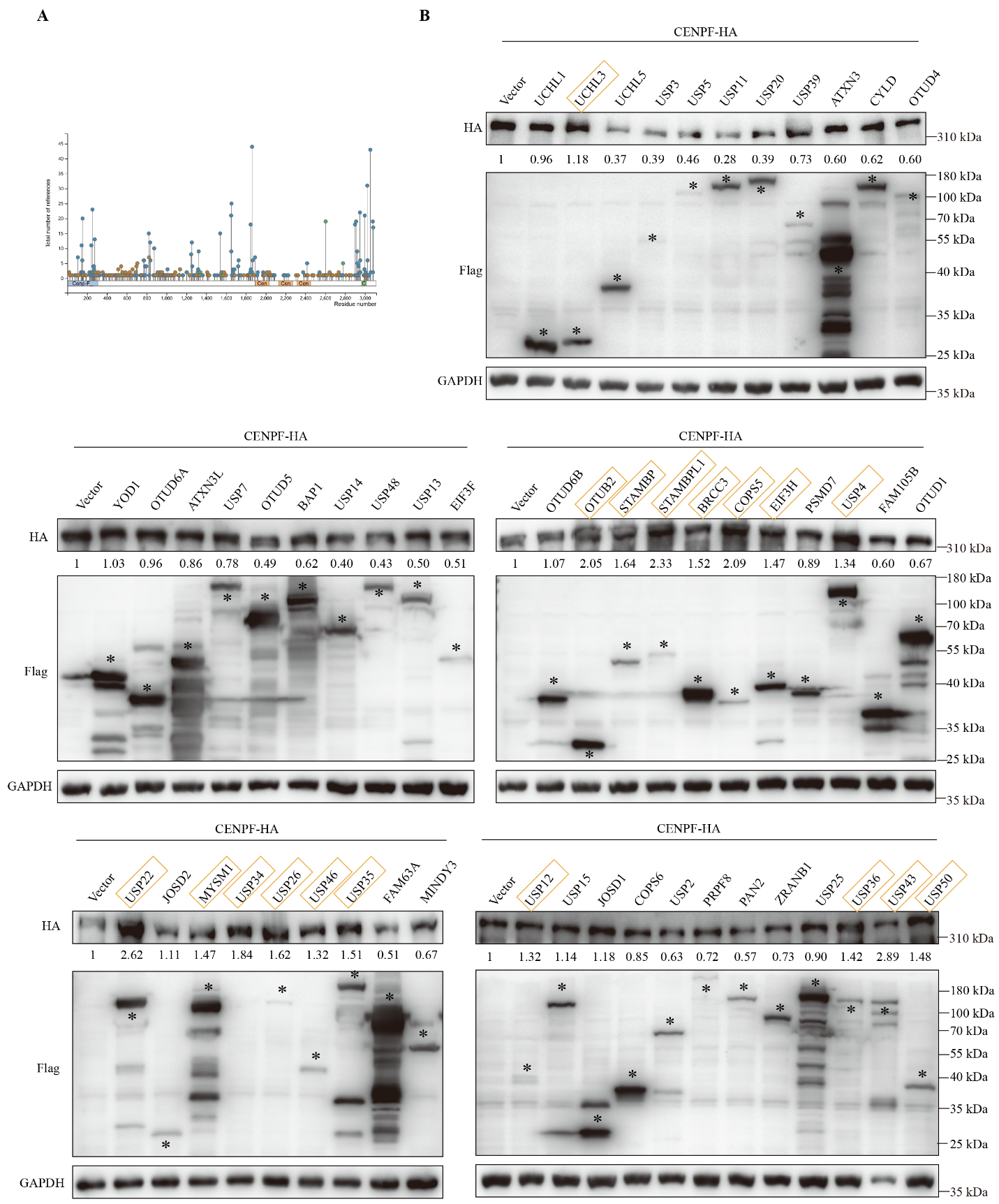


**Supplemental Figure 6. The DUBs library screened to find components that bolster CENPF protein expression. (A)** Graphic representation of the predicted modification sites of the CENPF protein. Blue circles indicate phosphorylation modification sites, while orange circles indicate ubiquitination modification sites. **(B)** After co-transfecting 293T cells with CENPF-HA and various DUBs-Flag plasmids, protein levels were analyzed via Western Blot.


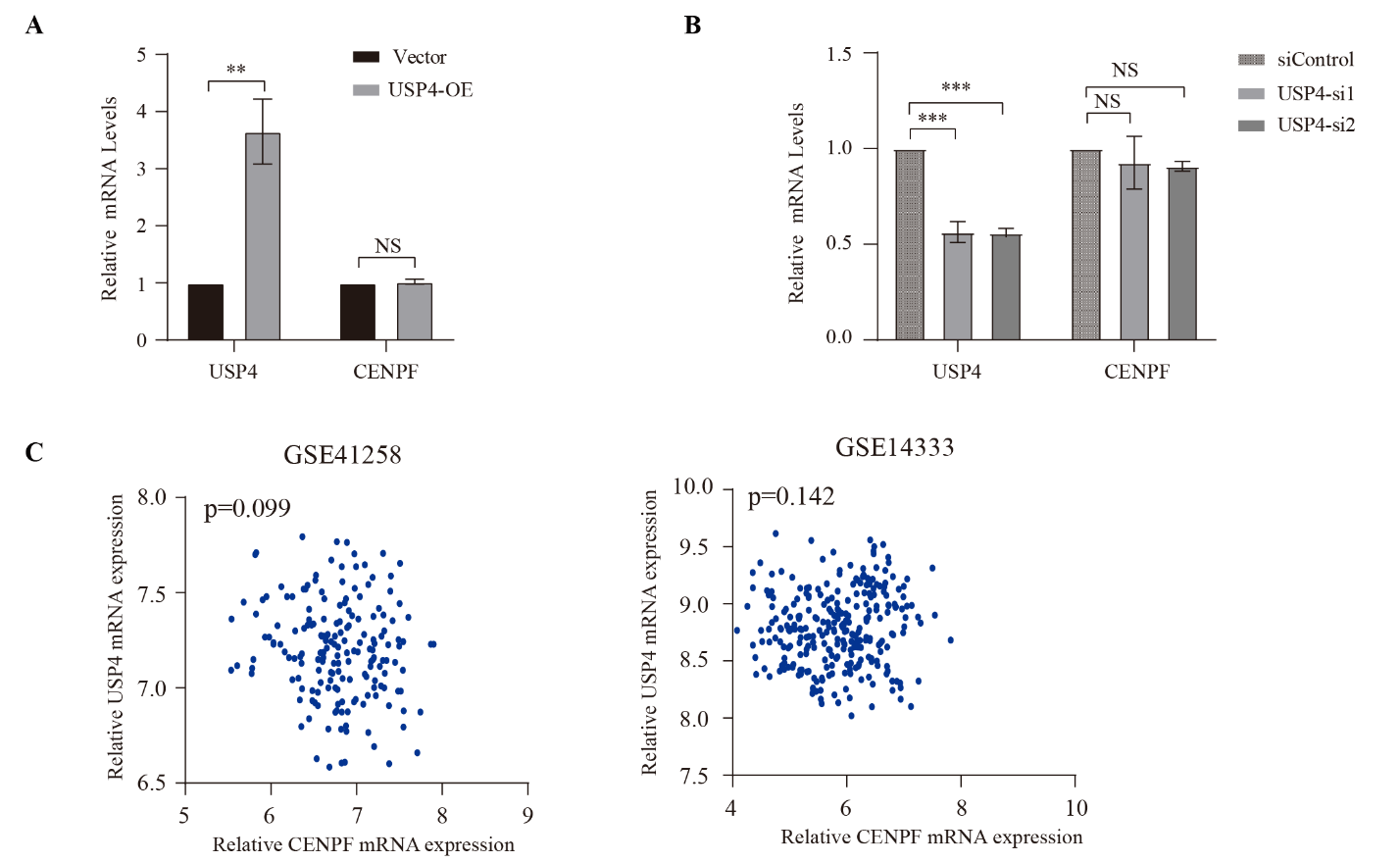


**Supplemental Figure 7. USP4 does not alter CENPF’s mRNA levels. (A)** Quantitative real-time PCR assessing CENPF mRNA levels in HCT116 cells following USP4 overexpression. Significance was gauged via Student’s t-test. **p < 0.01; NS. indicates non-significant. **(B)** CENPF mRNA levels in HCT116 cells, post USP4 suppression, were quantified using real-time PCR. Significance was gauged via Student’s t-test. ***p < 0.001; NS. indicates non-significant. **(C)** Analyzed correlations between the mRNA expression levels of USP4 and CENPF in CRC tissue samples from GEO datasets (GSE41258 and GSE41333). The Spearman’s rank correlation test was utilized.


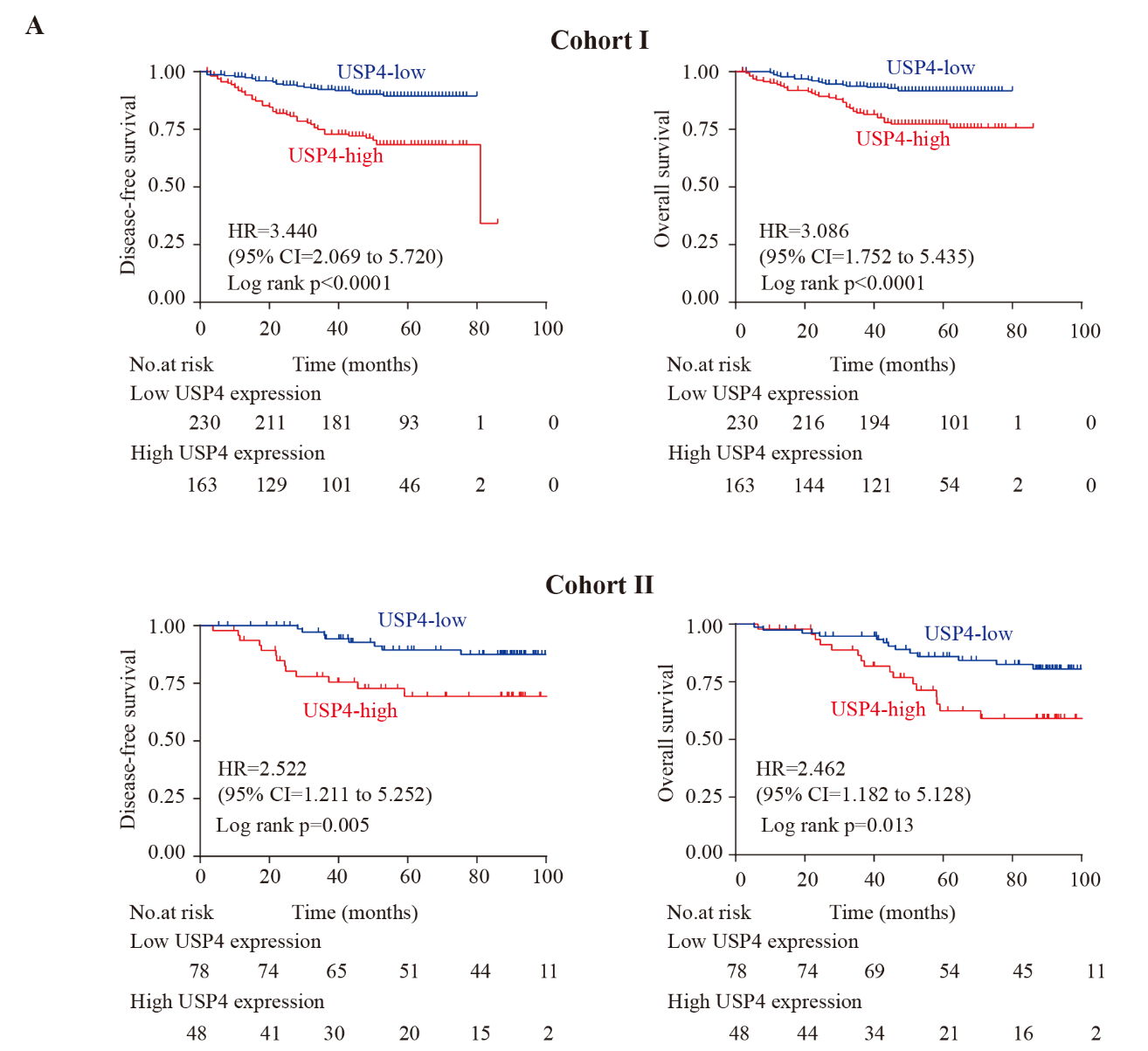


**Supplemental Figure 8. Based on USP4 protein expression and patient survival in Cohort I and Cohort II. (A)** CRC patients from stages I-IV were grouped into either USP4-high or USP4-low subgroups, utilizing the consistent cut-off value (100) for the USP4 IHC score from both cohorts. Kaplan-Meier survival curves for overall survival (OS) and disease-free survival (DFS) in CRC patients, based on USP4 protein expression subgroups in the cohorts I and II, are shown. Log-rank p-values and Hazard Ratios (HRs) are provided.


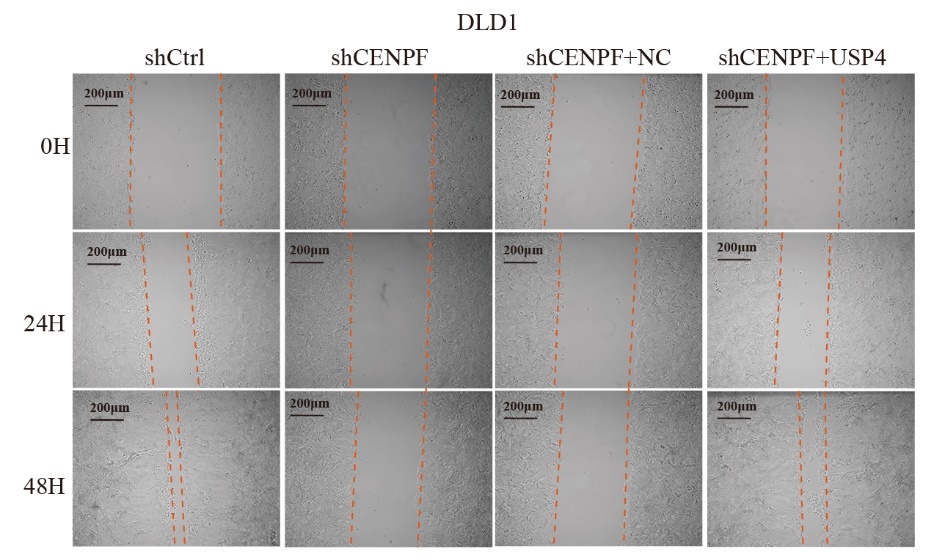


**Supplemental Figure 9. Representative images of the wound-healing migration assays in specific DLD1 cells.**


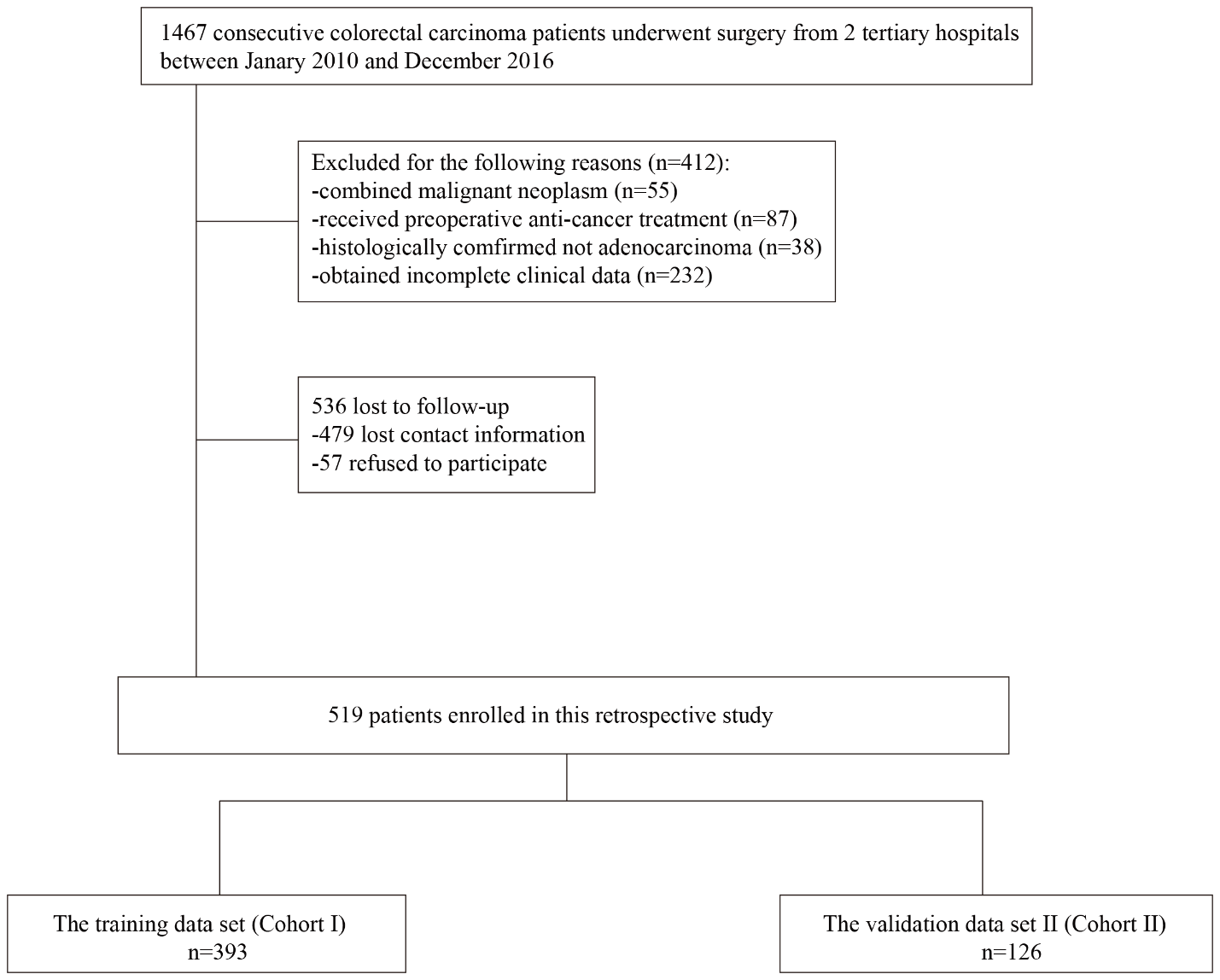


**Supplemental Figure 10.** **Diagram illustrating the selection process and criteria for patient inclusion in the study.**


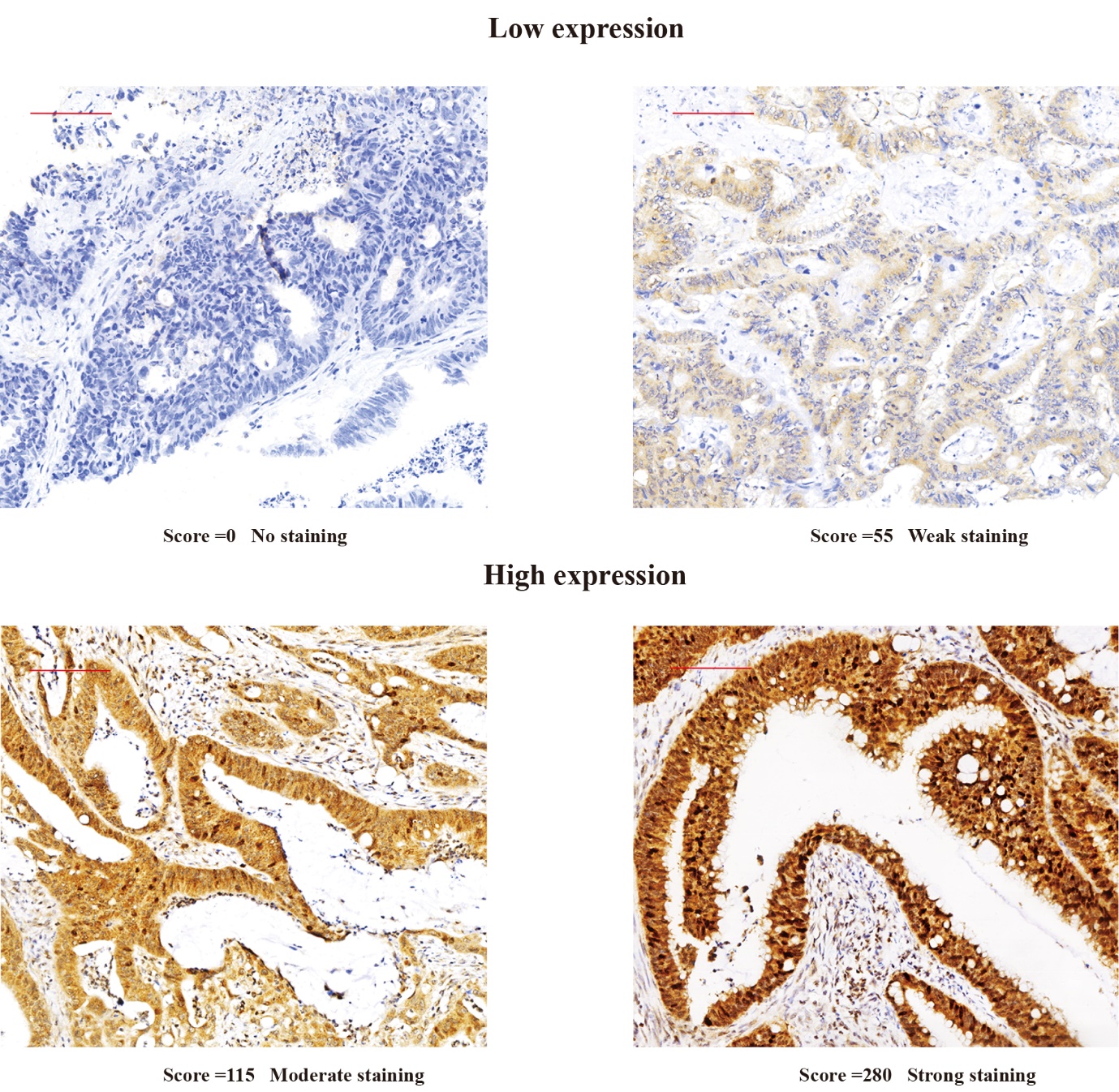


**Supplemental Figure 11. Immunohistochemistry scoring system for CENPF protein expression in colorectal specimens.**


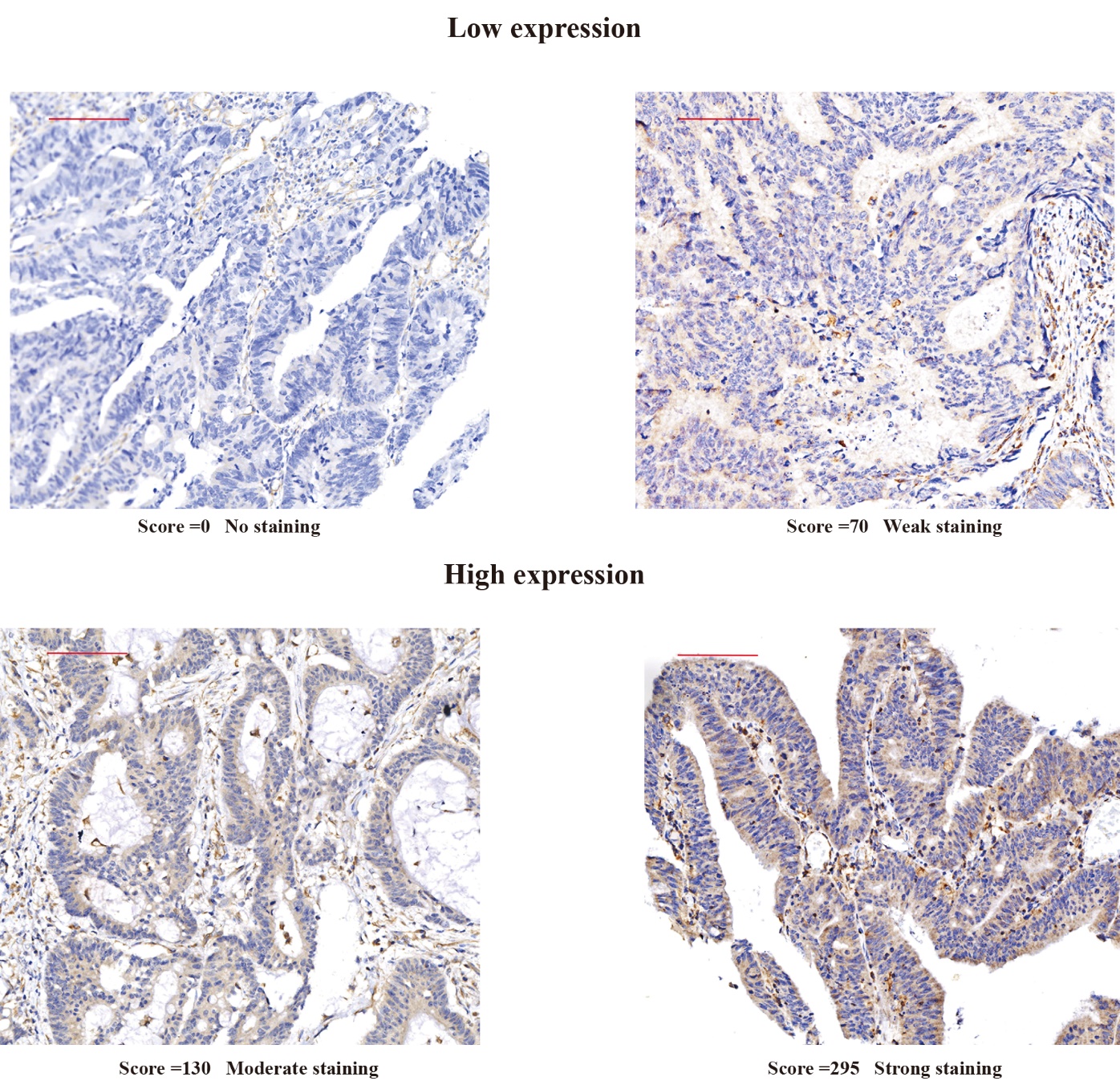


**Supplemental Figure 12.** **Immunohistochemistry scoring system for USP4 protein expression in colorectal specimens.**

**Full and uncropped western blots**


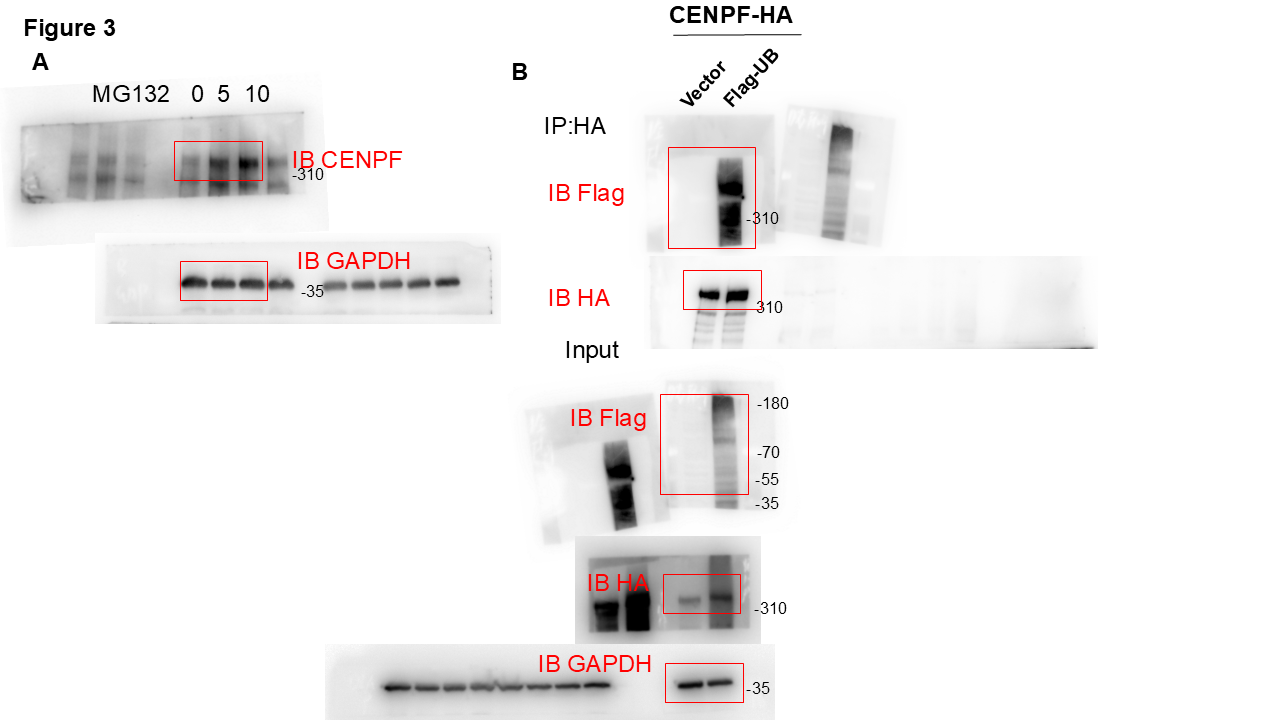


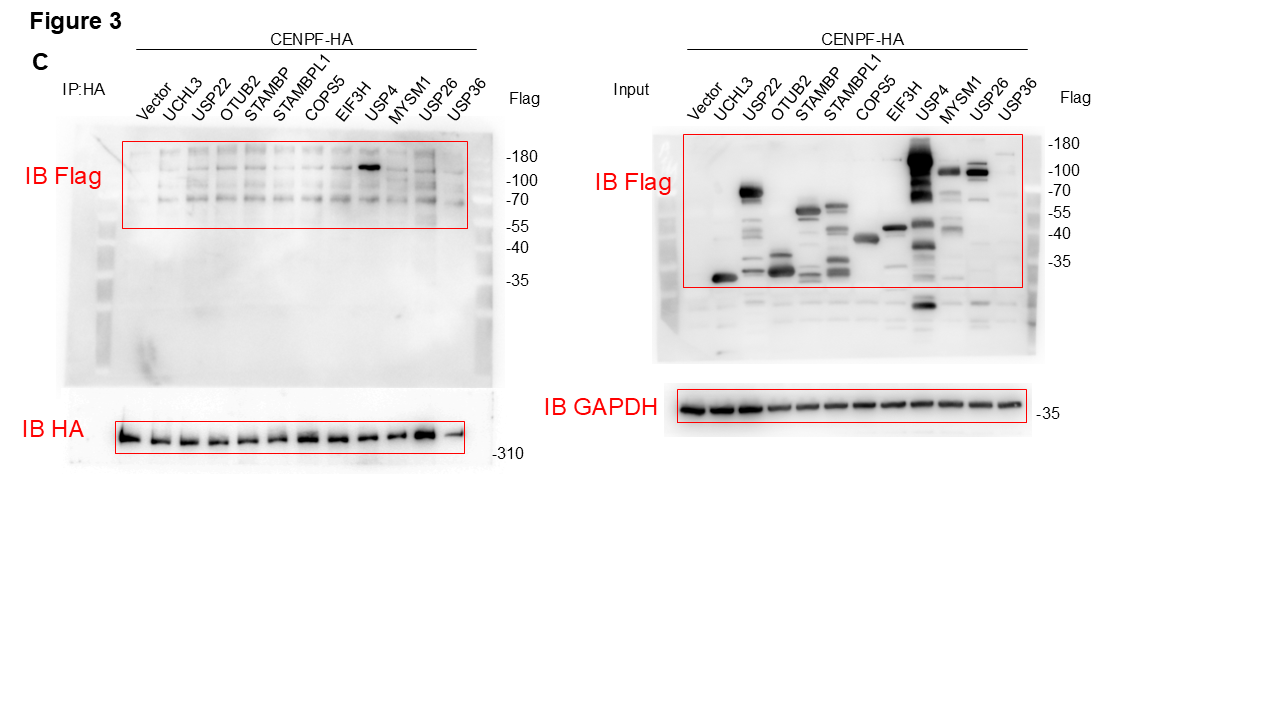


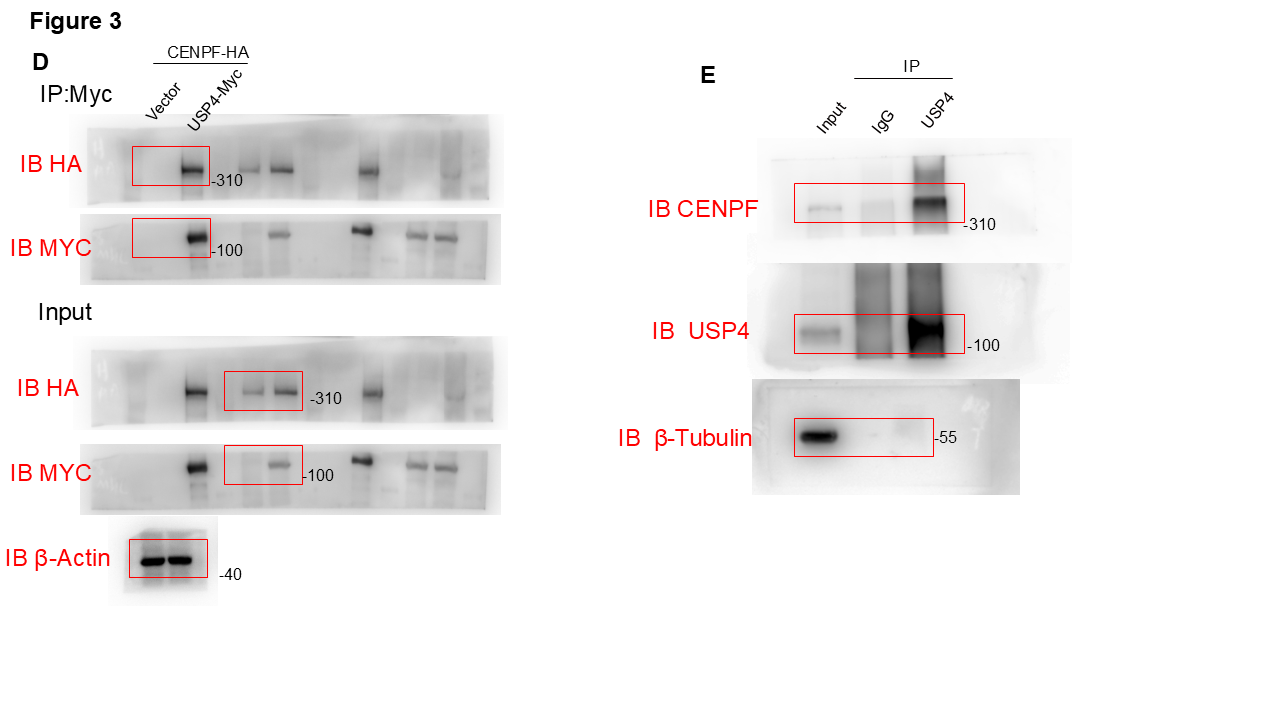

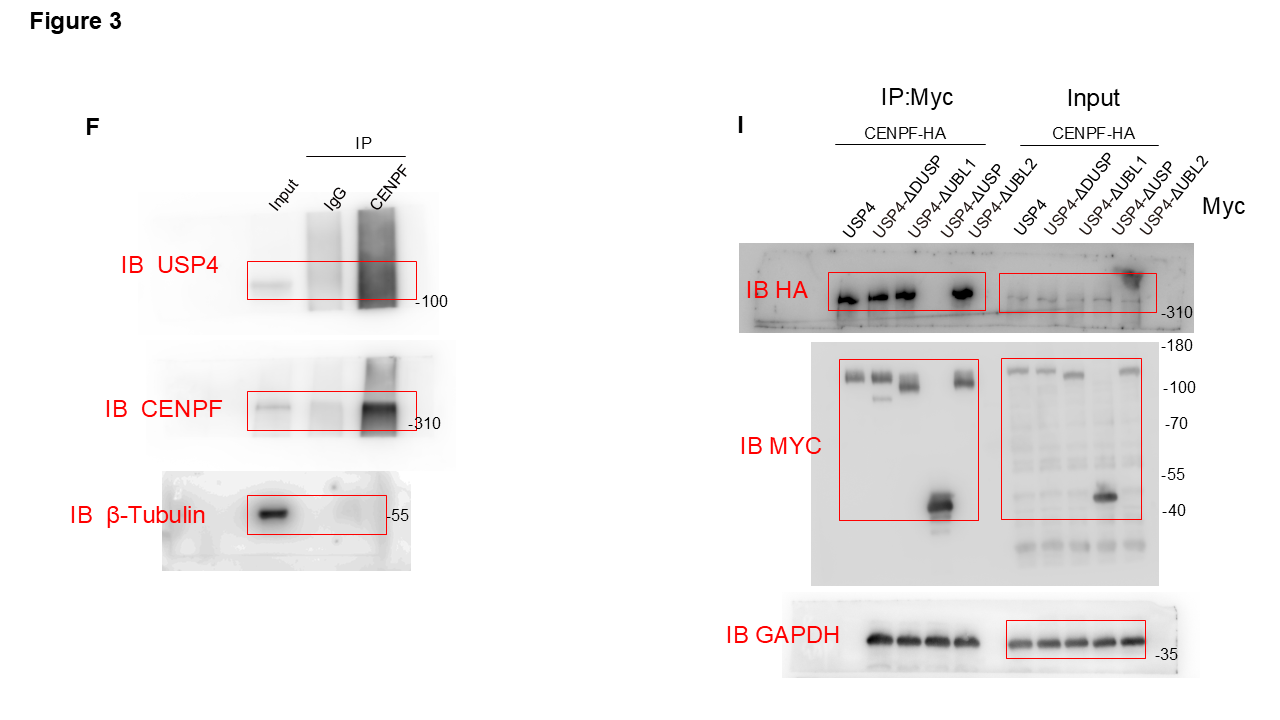

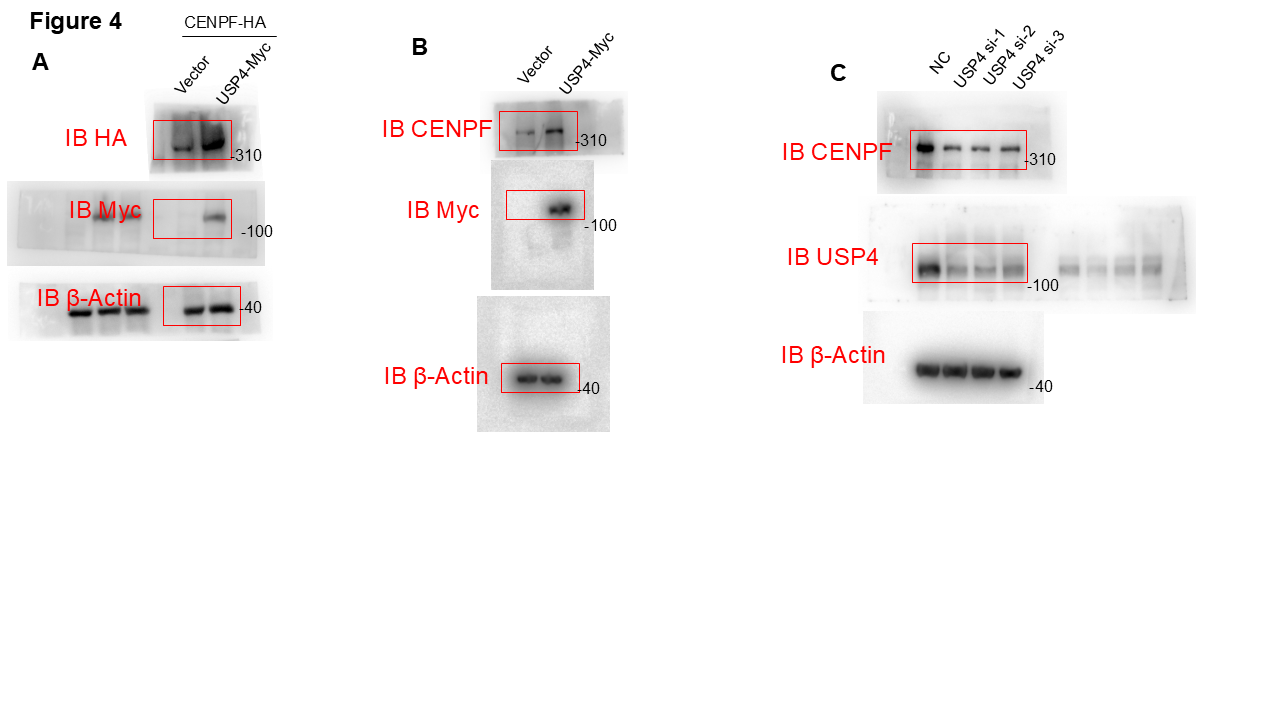

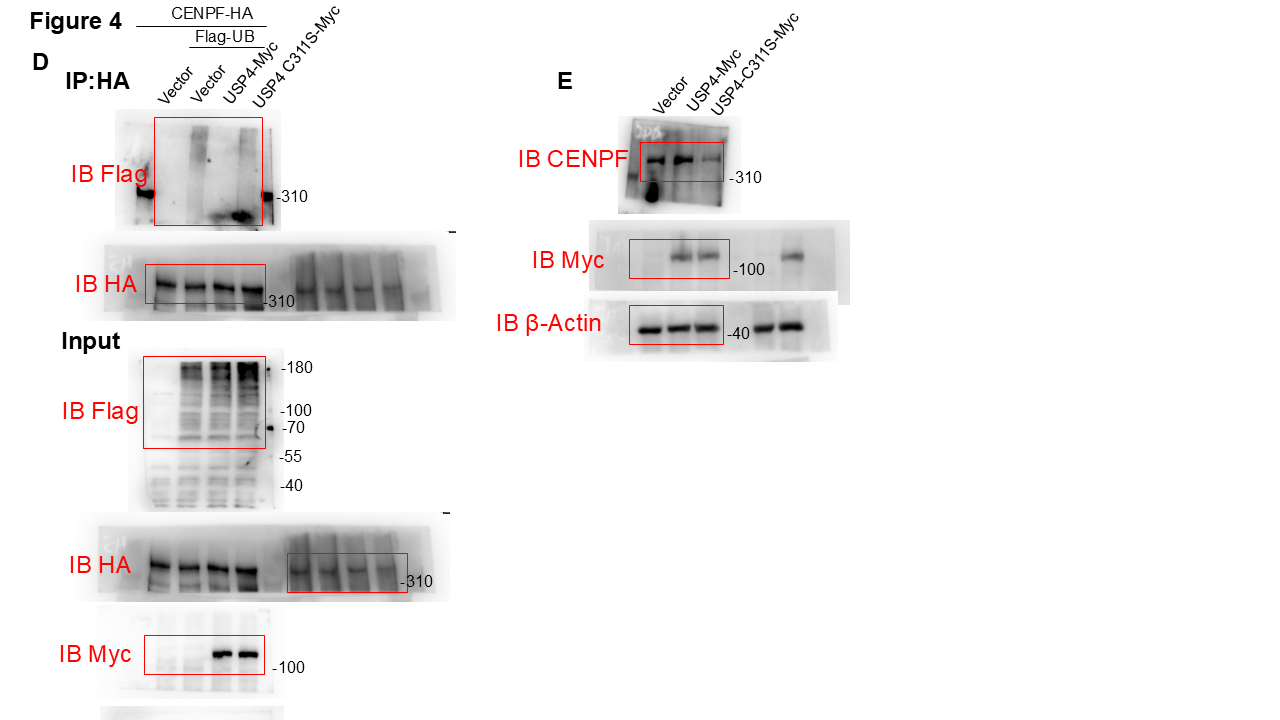

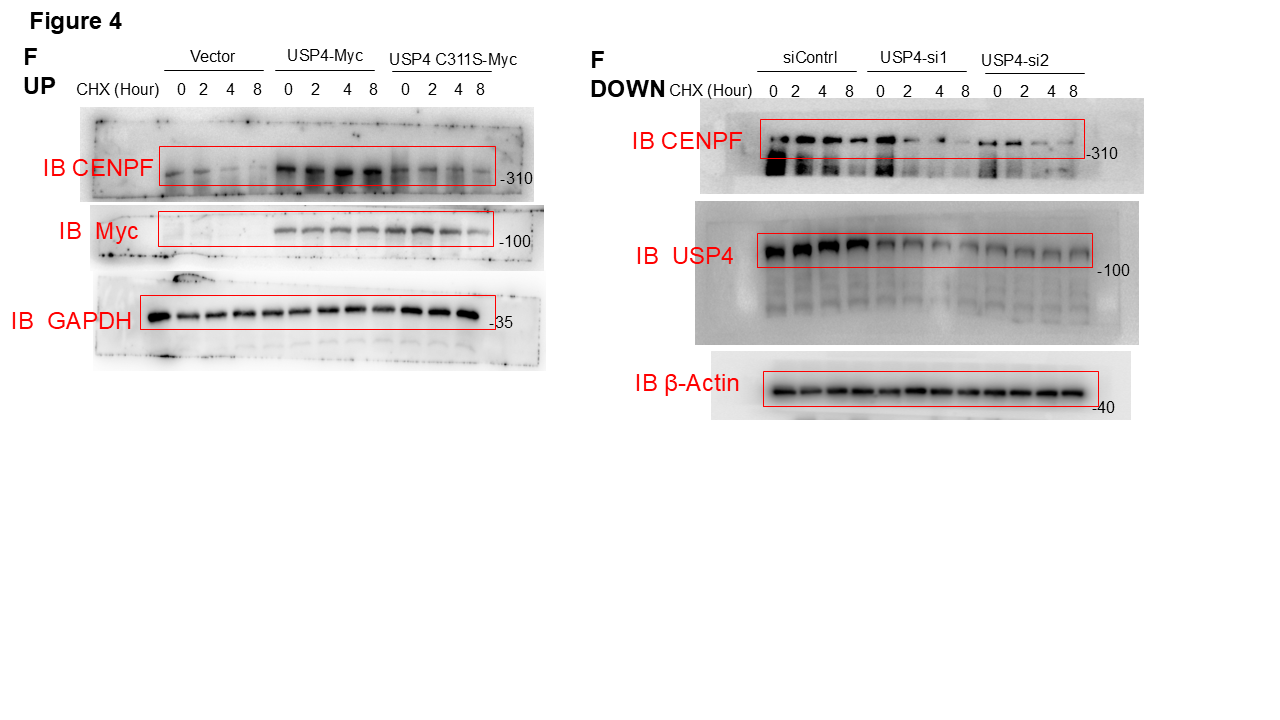

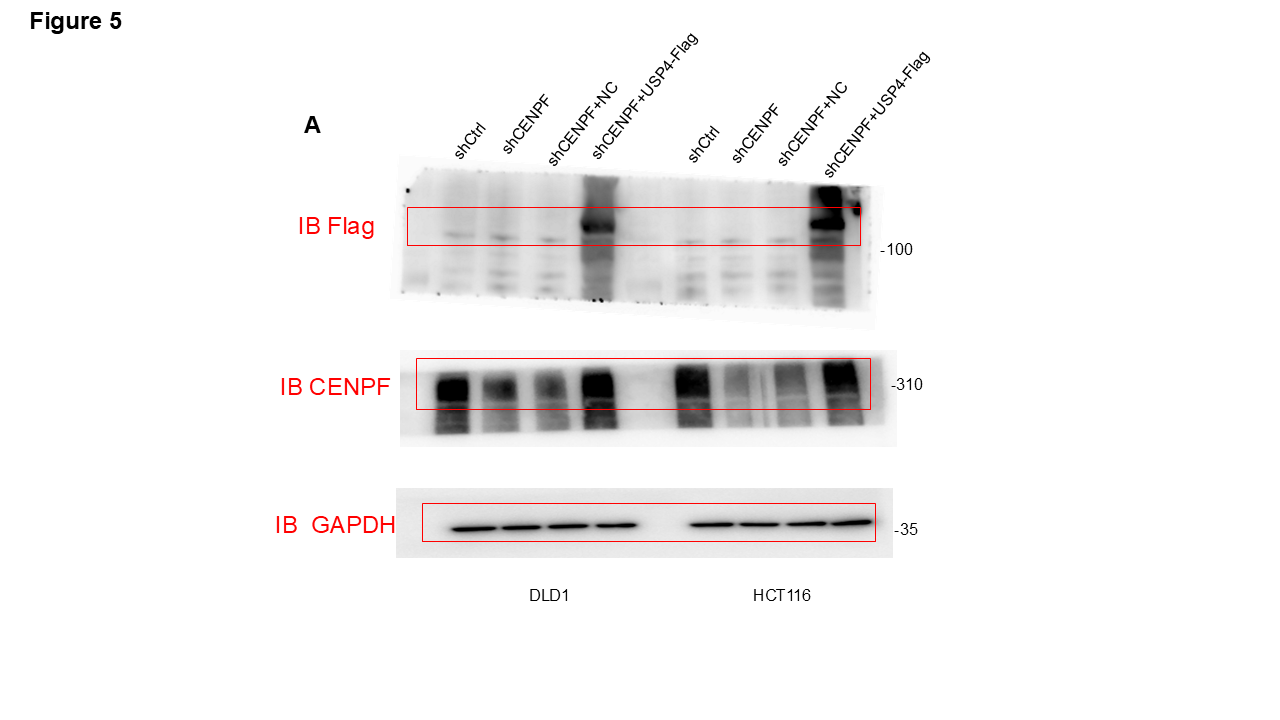

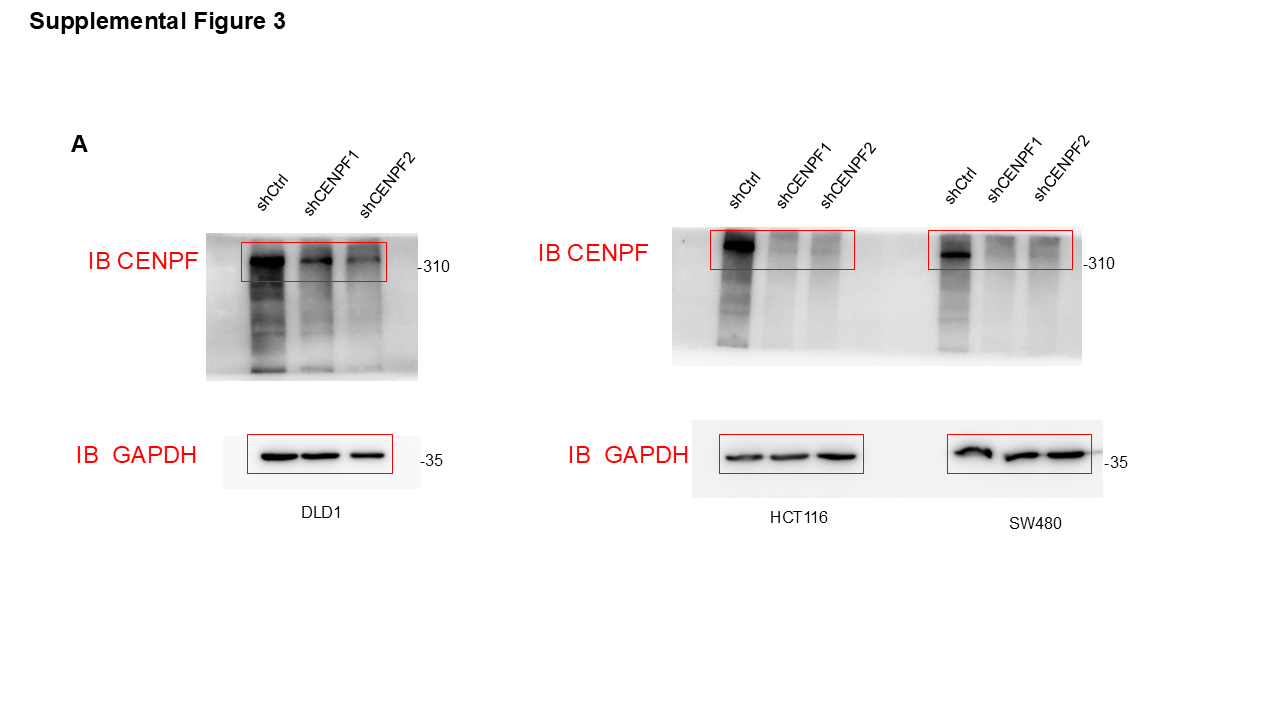

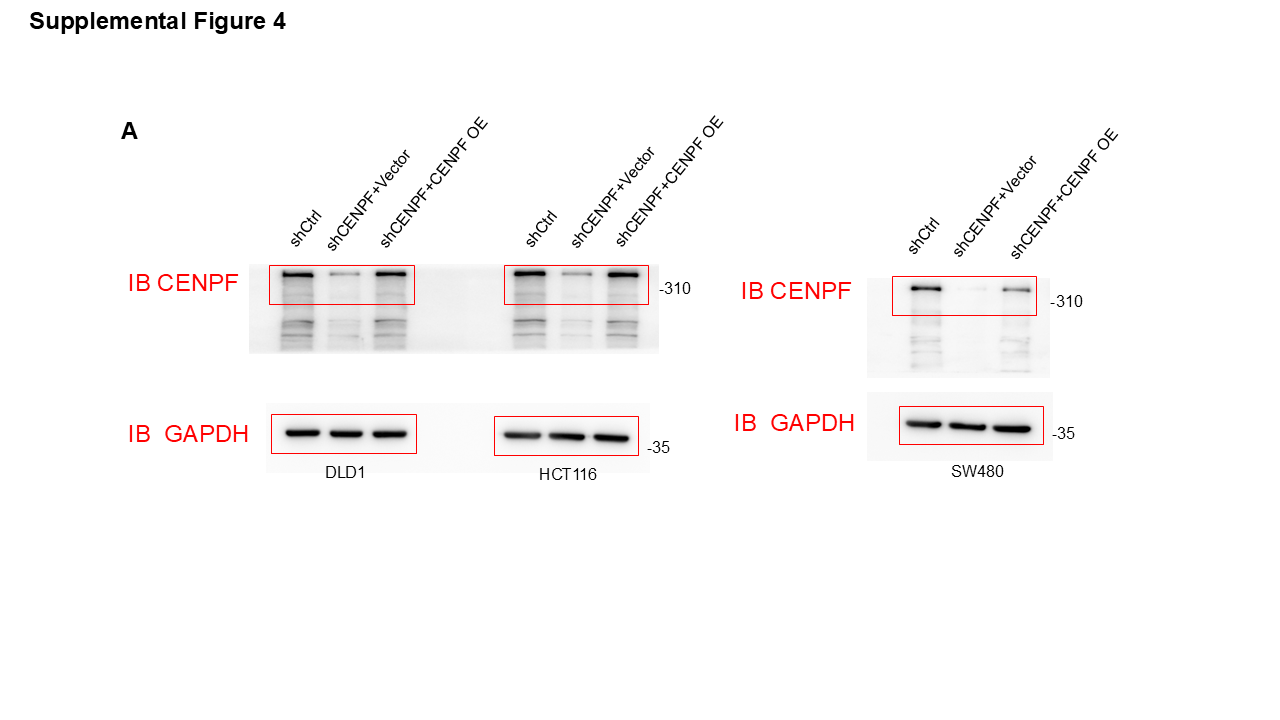

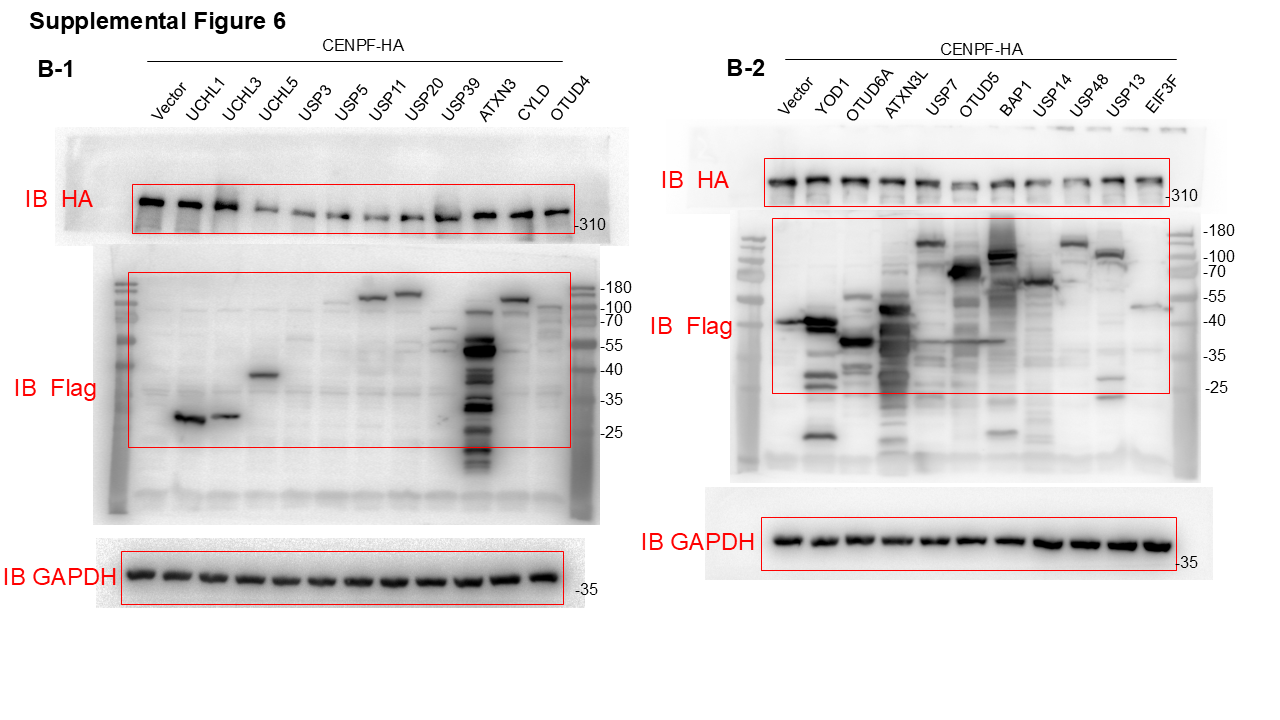

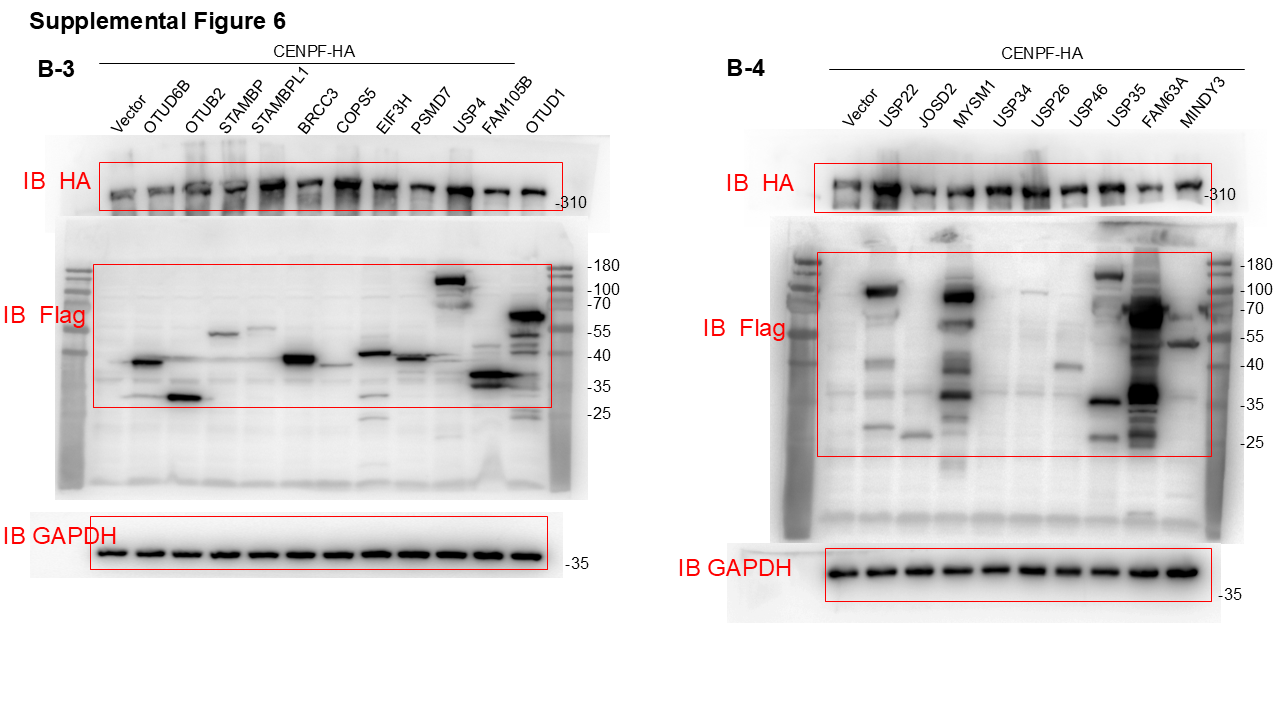

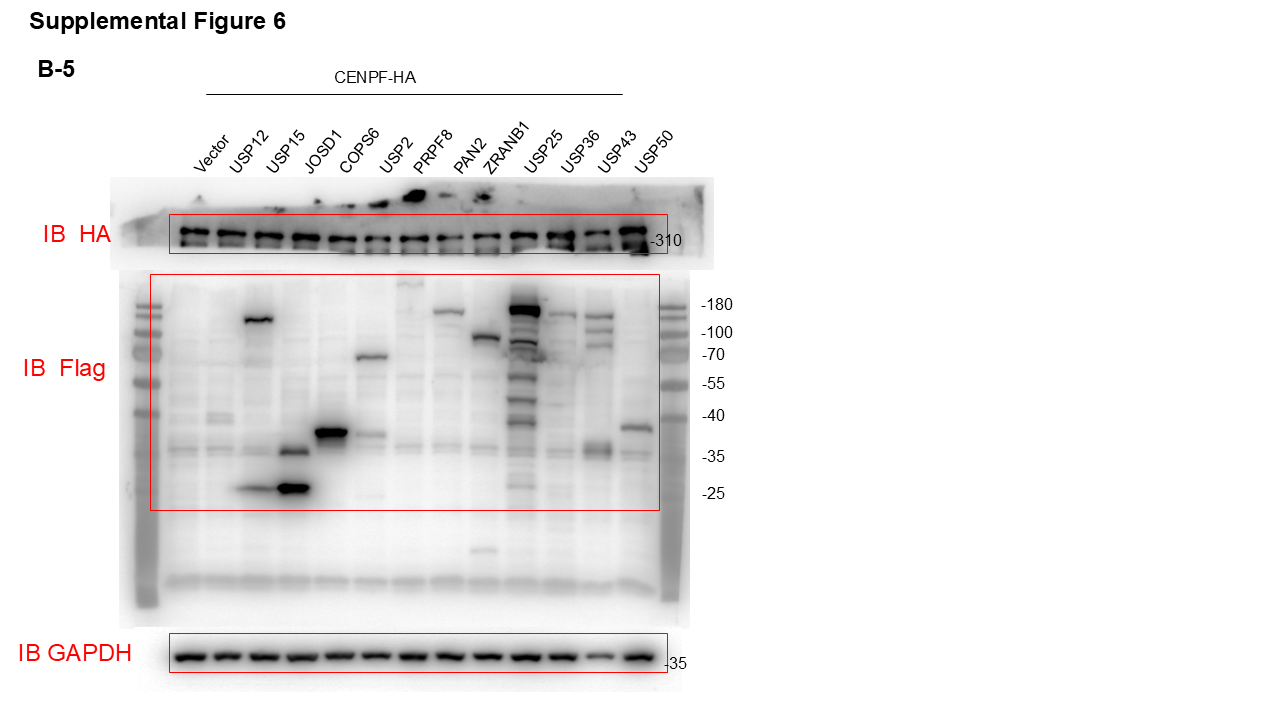

Supplement: Supplementary file 1 — Supplementary Material [file 41419_2025_7424_MOESM1_ESM.docx]
